# METTL1-mediated m^7^G tRNA modification drives papillary thyroid cancer progression and metastasis by regulating the codon-specific translation of TNF-α

**DOI:** 10.1038/s41419-025-07716-8

**Published:** 2025-05-14

**Authors:** Weiwei Li, Ruiwang Xie, Huaying Chen, Junyu Lin, Minjie Zhong, Junsi Zhang, Shengkai Zheng, Cen Jiang, Xiangjin Chen, Sunwang Xu

**Affiliations:** 1 Department of Thyroid and Breast Surgery, The First Affiliated Hospital, Fujian Medical University, Fuzhou, China; 2 Central Laboratory, Fujian Medical University Union Hospital, Fuzhou, China; 3 Department of Thyroid and Breast Surgery, National Regional Medical Center, Binhai Campus of the First Affiliated Hospital, Fujian Medical University, Fuzhou, China; 4 Fujian Provincial Key Laboratory of Precision Medicine for Cancer, Fuzhou, China

**Keywords:** Oncogenes, Oncogenesis

## Abstract

N7-methylguanosine (m^7^G) modification of transfer RNA (tRNA) is essential for the biological functions of tRNAs and has been found to play a regulatory role in a variety of human cancers. However, the biological function of METTL1-mediated m^7^G tRNA modification in papillary thyroid cancer (PTC) is unclear. Here, we found that METTL1 is significantly upregulated in PTC tissues compared to normal control tissues and is associated with poor PTC prognosis. Functional analysis confirmed that METTL1 promotes the proliferation and metastasis of PTC cells in a manner dependent on its tRNA methyltransferase activity. Mechanistically, METTL1 knockdown leads to a decrease in the abundance of certain m^7^G-modified tRNAs, which suppresses the m^7^G tRNA modification-mediated codon-specific translation of TNF-α. Furthermore, exogenous supplementation with TNF-α partially reversed the decrease in the proliferation and metastasis of PTC cells induced by METTL1 deletion. Positive correlations between METTL1, WDR4, and TNF-α expression, which affect the proliferation and metastasis of PTC, were confirmed via analysis of microarrays containing PTC tissues. These results demonstrate the oncogenic role of METTL1-mediated m^7^G tRNA modification in regulating codon-specific translation efficiency in PTC and suggest that targeting METTL1 may be a promising therapeutic approach for overcoming PTC progression by inhibiting PTC cell proliferation and metastasis.

## Introduction

Thyroid cancer ranks ninth in incidence rate among cancers worldwide [1, 2], and the incidence of thyroid cancer is increasing annually [3]. Papillary thyroid cancer (PTC) is the most common histologic type of thyroid cancer, accounting for more than 80% of all thyroid cancers [4], and is characterized by an increased tendency toward capsular infiltration and compression of surrounding organs and a high rate of metastasis to local lymph nodes [5]. Although PTC patients have excellent prognoses, with a 10-year survival rate higher than 90%, lymph node metastasis, capsular infiltration, extrathyroid invasion, and even distant metastasis are common. The initiators of PTC carcinogenesis have been reported to be single mutations, such as the BRAF V600E mutation, the KRAS S65N mutation, and the Ret proto-oncogene (RET)/PTC rearrangement [6]. However, many patients with PTC do not exhibit these somatic alterations [7], suggesting that certain molecular mechanisms that drive PTC tumorigenesis and progression independent of somatic mutations exist. Therefore, exploration of the molecular networks is urgently needed to reveal potential therapeutic targets to prevent PTC progression and metastasis.

In recent years, studies via epitranscriptomics have revealed the crucial role of multiple RNA posttranscriptional modifications in cancer [8, 9], with over 160 chemical modifications found in various RNAs [10–12]. For example, N6­methyladenosine (m6A) modification of messenger RNA (mRNA), one of the most intensively studied RNA modifications in cancer [13, 14], has been shown to have oncogenic effects on acute myeloid leukemia (AML) [15, 16], lung cancer [17], and liver cancer [18], which provides additional ideas for studying cancer progression at the posttranscriptional level. In addition, transfer RNAs (tRNAs), as important links between transcription and translation, have received increasing attention. Compared with mRNAs, tRNAs are more extensively modified [19]. These modifications are essential for stabilizing the structure and function of tRNAs so that they can further influence the accuracy and efficiency of protein translation [20]. Different kinds of tRNA modifications may be associated with mental retardation [21–23], cancer [24, 25], type 2 diabetes mellitus [26] and human mitochondrial diseases [27, 28]. However, the underlying molecular mechanisms by which tRNA modifications drive PTC progression and metastasis are poorly understood.

N7-methylguanosine (m^7^G) at position 46 of the tRNA nucleotide is one of the most prevalent tRNA modifications and is mediated mainly by the METTL1/WDR4 complex [29, 30]. METTL1 is an m^7^G catalytic enzyme that transfers other methyl groups to tRNA variable loops, whereas WDR4 mainly plays a role in stabilizing the catalase complex [29, 31]. This modification was previously reported in yeast [32], but recent studies have shown that METTL1/WDR4-mediated m^7^G tRNA modification is strongly linked with tumorigenesis and cancer progression in mammals [33–37]. However, the oncogenic function and potential mechanism of METTL1/WDR4-mediated m^7^G tRNA modification in PTC are unclear.

Here, we demonstrated that METTL1 promotes the proliferation and metastasis of PTC and drives its malignant biological behavior in a manner dependent on its tRNA methyltransferase activity. We also report that METTL1 specifically modulates the translation of tumor necrosis factor-α (TNF-α) via a tRNA m^7^G modification-mediated codon-specific translation approach, which has not yet been reported. These findings will be helpful in clarifying the proliferation- and metastasis-driving functions of epigenetic alterations in tRNA modification and supporting the rationale for the development of a novel therapeutic strategy for targeting PTC progression and metastasis.

## Materials and methods

### Cell lines

Human PTC cell lines TPC-1, IHH4, BCPAP, and K1 were obtained from the Cell Bank of Type Culture Collection of the Chinese Academy of Science, and the human embryonic kidney 293T (HEK293T) cell was obtained from the American Type Culture Collection. All cells were cultured in DMEM medium, which was supplemented with 10% fetal bovine serum (FBS) (Gibco) in a humidified atmosphere of 5% CO_2_ at 37 °C, and were tested monthly for mycoplasma and were negative.

### Cell transfection

METTL1 targeting short-harpin RNA and non-specific control shRNA (shNC) used in this study were obtained from Beijing Tsingke Biotech. The coding sequence of METTL1 was cloned into the pCDH-CMV-MCS-EF1-Puro vector. To generate and infect lentiviruses, HEK293T cells at 80–90% confluency were cotransfected with 1.76 μg of knockdown or overexpression plasmid, 1.32 μg of psPAX, and 0.88 μg of pMD2.G in 6 mm dishes with 12 μl of polyethylenimine. After transfection for 6 h at 37 °C, the medium was replaced, and the lentivirus-containing medium was harvested 72 h later. Then, the PTC cells were infected by the lentivirus supplemented with polybrene for 24 h, and the puromycin-resistant cells were harvested as the stable transfected cell lines. The sense sequence of shRNAs was: METTL1-sh1, 5’-GGTGTATACCATAACCGATGT-3’; METTL1-sh2, 5’-CCCACATTTCAAGCGGACAAA-3’.

To METTL1 and TNF-α overexpression plasmids, the full-length open reading frames (ORFs) of the human METTL1 (NM_005371.6) and TNF-α gene (NM_000594.4) were separately cloned into the pCDH-CMV-MCS-EF1-Puro vector. The METTL1 catalytic inactive mutant (aa160-163, LFPD to AFPA) was generated using the Q5 Site-Directed Mutagenesis Kit (New England Biolabs). The TNF-WT and MUT cDNA were obtained from Fuzhou Sunya Biotechnology Co.

### Cell proliferation assay

Cell proliferation was assessed by the Cell Counting Kit-8 (CCK-8) (Yeasen) method. PTC cells in single-cell suspension were inoculated in 96 wells at 1000 cells/well with 100 μl of medium. The 10 μl of CCK-8 solution was added to the cells at the indicated time points, and the cells were incubated for 1 h at 37 °C. The reaction products were quantified at an absorbance of 450 nm using a Synergy 2 enzyme labeler (Biotek).

### Colony formation assay

For colony formation assay, PTC cells were seeded in 6-well plates at a density of 250 cells/well and incubated with 2 ml of culture medium per well for culturing until colonies were visible. Then, clones were fixed with 10% paraformaldehyde and stained with 0.1% crystal violet.

### Migration and invasion assay

For the migration assay, PTC cells were starved without FBS for 4 h, then 40000 TPC-1, BCPAP, K1 cells, and 100000 IHH4 cells were resuspended in 100 μl of culture medium without FBS and cultured in the upper chamber of an uncoated transwell insert in a 24-well plate (Corning). Six hundred microliters of culture medium containing 10% FBS was added to the lower chamber as a chemoattractant to induce cell migration. For the invasion assay, the upper chamber of the transwell inserts was pre-coated with 100 μl of 300 μg/ml Matrigel (Corning), and the cells were cultured in the migration assay. After 24 h incubation at 37 °C, all cells were stained with 0.1% crystal violet, and the non-migrated or non-invaded cells were gently removed with a cotton swab. Migrated or invaded cells in five fields of view were photographed and counted under an inverted microscope.

### Cell cycle assay

For the cell cycle assay, PTC cells seeded in 6-well plates (70–80% confluence) were harvested by treatment with EDTA-free trypsin. After washing twice with PBS, cells were resuspended and fixed in cold absolute ethanol overnight at −20 °C. Cell-cycle analysis was performed according to the manufacturer’s instructions of the Cell Cycle Staining Kit (Multi Sciences) with an Accuri C6 flow cytometer (BD Biosciences). The cell cycle analysis was completed by flowjo software.

### In vivo xenograft model

Male Balb/c immunodeficient mice (15–20 g, 4 weeks old) were purchased from SLAC Laboratory Animals, Shanghai. Mice were randomly assigned to a knockdown group and a control group. Blinding was not performed during animal experimental procedures and outcome assessment. The number of animals used was based on prior studies in similar experimental models [7], and adherence to the principle of reducing animal numbers while maintaining scientific rigor. For Subcutaneous Xenograft, 1 × 10^6^ METTL1-depleted cells or control K1 cells with 100 μl PBS were injected subcutaneously into mice. Seven days after subcutaneous inoculation, the length and width of the tumors (in mm) were measured for the first time and then every 4 days thereafter. The tumor volume was calculated using the formula (length × width^2^)/2. At the end of the experiment, mice were euthanized, tumors were removed, and the weight of the tumors was measured.

For rescue assay, 1 × 10^6^ METTL1-depleted cells or control K1 cells with 100 μl PBS were injected subcutaneously into mice. One week after injection, the mice were randomized into two groups, TNF-α recombinant protein (50 μg/kg) or vehicle was injected intraperitoneally into the xenograft mice. At the end of the experiment, mice were euthanized, tumors were removed, and the weight of the tumors was measured. In vivo studies were conducted by the National Institutes of Health Guidelines for the Care and Use of Laboratory Animals, and the study procedures were approved by the Ethics Committees of The First Hospital of Fujian Medical University.

### Inguinal lymph node metastasis model

Male Balb/c immunodeficient mice (15–20 g, 4 weeks old) were purchased from SLAC Laboratory Animals, Shanghai. For in vivo lymph node metastasis, 2 × 10^5^ METTL1-depleted cells or control K1 cells with 30 μl PBS were injected into the mouse footpads. After 4 weeks, the footpad tumors metastasized to the inguinal lymph nodes, and the footpad tumors and inguinal lymph nodes were excised for analysis.

### RNA-seq analysis

Transcriptome sequencing was performed by Sangon Biotech (Shanghai, China). Briefly, Total RNA was extracted using Trizol reagent, and genomic DNA contamination was removed by RNase-free DNase I. RNA integrity was evaluated with a 1.0% agarose gel. Then the quantity and quality of RNA were detected using a Qubit 2.0 Flurometer (Invitrogen) and a NanoPhotometer spectrophotometer (IMPLEN). The high-quality RNA samples were used for library preparation and sequencing. The quality of sequenced data was evaluated using FastQC (version 0.11.2).

### Tandem mass tag quantitative proteomic analysis

Proteomic analysis was performed by Luming Biotechnology Co. (Shanghai, China). Briefly, proteins were extracted from the PTC cell sample, and protein concentration was determined by BCA assay (Thermo Scientific). Trypsin was added to protein samples for enzymatic digestion. Finally, the protein samples were labeled with peptides using TMT reagent and separated by reverse-phase chromatography. Proteins were further classified via gene ontology (GO) and gene set enrichment analysis (GSEA) pathway analysis.

### RNA extraction and real-time quantitative PCR

Total RNA was extracted from PTC cells using the Total RNA Rapid Extraction Kit (Sparkjade) following the manufacturer’s protocol, and 1 μg of total RNA was reverse transcribed to cDNA using the HiFi II 1st strand cDNA synthesis kit (Yeasen). Real-time quantitative PCR was performed on a CFX96 Real-Time PCR System (Bio-Rad, Hercules, CA, USA). The relative mRNA expression was calculated with the 2^−ΔΔCt^ method and normalized to the internal reference gene GAPDH. The primers for real-time PCR as follows: METTL1, forward 5’-GGCAACGTGCTCACTCCAA-3’, reverse 5’-CACAGCCTATGTCTGCAAACT-3’; TNF-α, forward 5’-AACCTCCTCTCTGCCATCAA-3’, reverse 5’-GGAAGACCCCTCCCAGATAG-3’; TIM3, forward 5’-CTGCTGCTACTACTTACAAGGTC-3’, reverse 5’-GCAGGGCAGATAGGCATTCT-3’; OXR1, forward 5’-TTCGACCAAACCTAAGTGATCCC-3’, reverse 5’-GGGGTGTCTAAACCTGTCATTG-3’; NCEH1, forward 5’-TGTTGTACGGGCCACAAAGTA-3’, reverse 5’-GCAGGATTGGGGTGTTCACAT-3’; IL33, forward 5’-CAAAGAAGTTTGCCCCATGT-3’, reverse 5’-TTTCAGTGAAGGCCTTTTGG-3’; CCL2, forward 5’-CCCCAGTCACCTGCTGTTAT-3’, reverse 5’-AGATCTCCTTGGCCACAATG-3’; NF-κB, forward 5’-GAAGCACGAATGACAGAGGC-3’, reverse 5’-GCTTGGCGGATTAGCTCTTTT-3’; MMP9, forward 5’-AGACCTGGGCAGATTCCAAAC-3’; reverse 5’-CGGCAAGTCTTCCGAGTAGT-3’; CXCL1, forward 5’- AGGGAATTCACCCCAAGAAC-3’, reverse 5’- TAACTATGGGGGATGCAGGA-3’; PD-L1, forward 5’- GCTGCACTAATTGTCTATTGGGA-3’, reverse 5’- AATTCGCTTGTAGTCGGCACC-3’; GAPDH, forward 5’- GGTGTGAACCATGAGAAGTATGA-3’, reverse 5’- GAGTCCTTCCACGATACCAAAG-3’.

### Western blot

Western blot was performed using standard procedures. Total proteins were extracted from PTC cells using RIPA lysis buffer (Meilunbio), and were quantified using a Micro BCA protein Assay Kit (Thermo Fisher Scientific). 20 μg of total protein was electrophoresed through a 10% SDS polyacrylamide gel and then transferred to PVDF membranes (Millipore). The membranes were blocked in 5% skim milk for 1 h at room temperature and then incubated with primary antibodies at 4 °C overnight. Next, the membrane was incubated with a suitable horseradish peroxidase (HRP) coupled secondary antibody for 1 h at room temperature. And the signals were detected using the ECL Kit (Meilunbio) by the ChemiDoc-MP imager (Bio-Rad). Using β-actin as a control for whole-cell lysin. Relative protein expression was calculated by Image J software and normalized to the expression of β-actin. The antibody against METTL1 (#ab271063, dilution 1:1000), β-actin (#ab8226, dilution 1:1000), DDDDK tag (#ab205606, dilution 1:10,000) and TNF-α (#ab215188, dilution 1:1000) were purchased from Abcam, puromycin (ZMS1016, dilution 1:10,000) was purchased from Sigma-Aldrich., p110α (Cat. #4249, dilution 1:1000), p85 (Cat. #4257, dilution 1:1000), Akt (Cat. #4691, dilution 1:1000) were purchased from Cell Signaling Technology.

### Enzyme-linked immunosorbent assay

PTC cells stably transfected with METTL1-shRNAs (METTL1-sh) or control shRNA (shNC), METTL1-WT, METTL1-MUT, or METTL1-EV were seeded in 6-well plates (5 × 10^5^ cells per well) and incubated with 2 ml of cultured medium for 24 h. Then, the supernatants were collected and subjected to ELISA with a Human TNF-α High Sensitivity ELISA Kit (Multi Science) according to the manufacturer’s instructions.

### Puromycin intake assay

PTC cells were transiently transfected with METTL small interfering RNA (siRNA) and NC (siNC) oligos using Lipofectamine 3000 (Thermo Fisher Scientific). Next, cells were treated with 10 μg/ml puromycin for 30 min and lysed with RIPA to extract protein. Western blot assay performed to detect the adulterated puromycin. The sense sequence of siRNAs was: METTL1-si1: 5’-GAUGGACUGGUCUGAGCUATT-3’; METTL1-si2: 5’-GGUGAAGGUCUCAGACUAUTT-3’.

### m^7^G site tRNA reduction and cleavage sequencing (TRAC-seq)

TRAC-seq was performed as previously described [38]. TRAC-Seq was performed by CloudSeq Biotech Inc. (Shanghai, China). Briefly, Total RNA was collected from the sample, and the small RNA fraction (<200nt) was separated from the total RNA using the miRNeasy Mini Kit (Qiagen). RNA fragments were treated with the demethylase AlkB, reduced with sodium borohydride, and then cleaved in 1 M aniline. According to the manufacturer’s instructions, use the GenSeq Small RNA Library Prep Kit (GenSeq, Inc.) to construct RNA libraries. The BioAnalyzer 2100 System (Agilent Technologies, USA) was used for library quality control and quantification. High-throughput sequencing was performed using an Illumina Nova sequencer.

### Clinical examples

A total of 48 cases of PTC tumor and adjacent tissues were collected from the Department of Thyroid and Breast Surgery, the First Affiliated Hospital of Fujian Medical University. The research project was approved by the Ethics Committee of the First Affiliated Hospital of Fujian Medical University. Before the study, all patients signed the consent of informed consent.

### Immunohistochemistry staining analysis

IHC staining was performed as described as we described previously [39]. Briefly, the PTC tumor samples were stained with METTL1 (#ab271063, dilution 1:300, Abcam), WDR4 (#ab169526, dilution 1:200, Abcam), TNF-α (YT4689, dilution 1:100, Immunoway). The staining (defined as histoscore) was scored as the intensity of positive staining ((0 = none; 1 = weak; 2 = moderate; and 3 = strong) multiplied by the proportion of positive staining (0 = none; 1 = 1–10%; 2 = 11–50%; 3 = 51–80%; 4 = 81–100%). Samples with a histoscore of more than or equal to 4 were considered to be high, and less than 4 were considered to be low. These scores were independently determined by two pathologists.

### Statistical analysis

All data in this study were obtained from three independent experiments and presented as mean ± SD. Normality of data distribution was assessed using the Shapiro–Wilk test. For normally distributed measurement data, a two-tailed unpaired or paired Student’s *t*-test was used to analyze the two groups' comparison, and a One-way ANOVA test was used for three or more groups. For non-parametric data, Mann–Whitney *U*-test (data with abnormal distributions) was applied. The chi-square test was used for categorical data. The Kaplan–Meier method and log-rank test were used to estimate the survival probabilities. Cox proportional hazards regression model was performed in univariate and multivariate analyses to determine the prognostic factors for PTC patients. Pearson correlation coefficient was used to analyze the correlation between METTL1, WDR4, and TNF-α protein expression. The differences were considered statistically significant if *P* < 0.05, and indicated by, ^*^P < 0.05, ^**^P < 0.01, ^***^P < 0.001; or ns, not significant.

## Results

### METTL1 expression is high in PTC, and this phenotype is associated with a poor prognosis

**Fig. 1:**
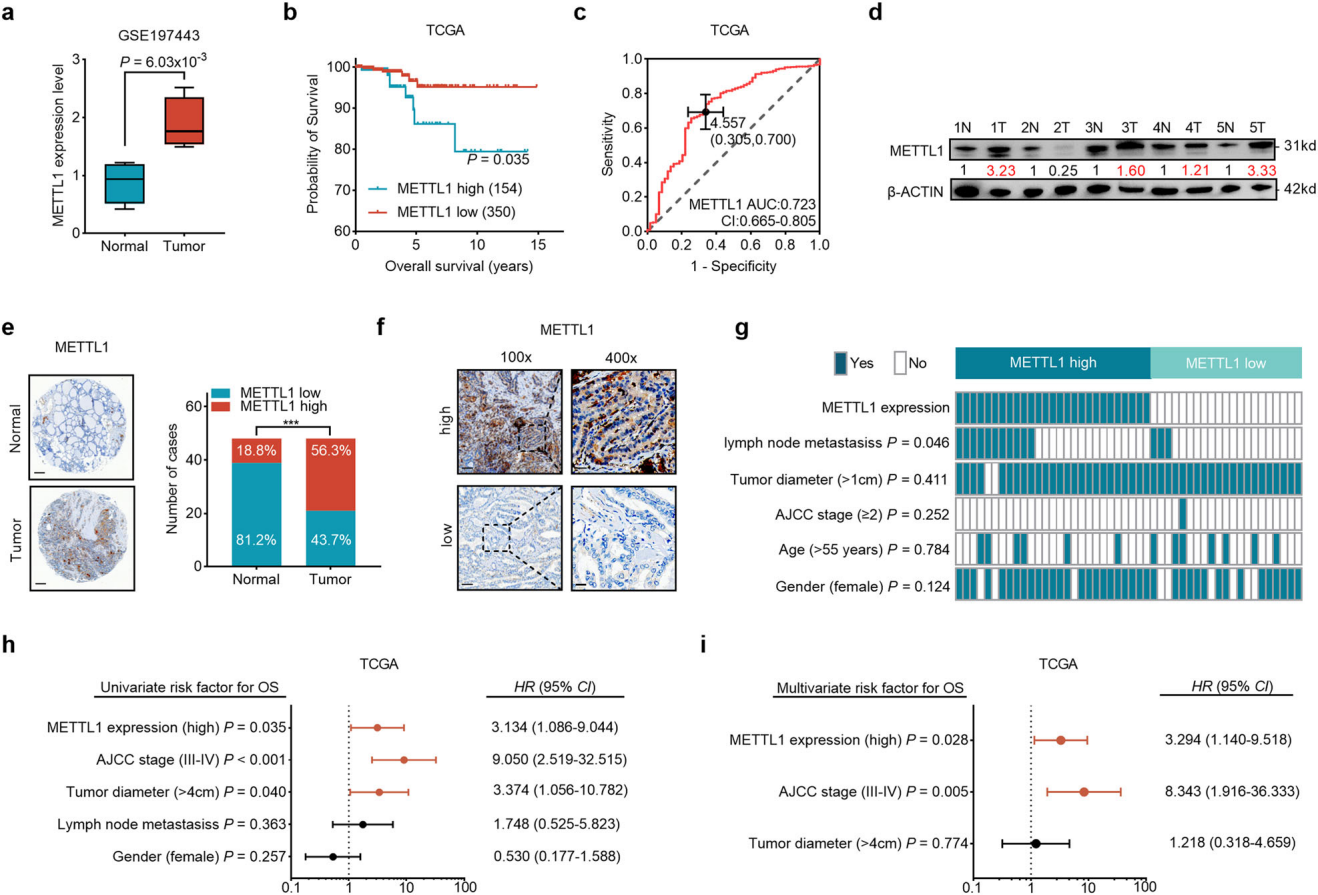
METTL1 expression is high in PTC, and this phenotype is associated with a poor prognosis. **a** METTL1 mRNA levels in PTC tumorous tissues and adjacent normal tissues in a GEO dataset, GSE197443. **b** OS was compared between PTC patients with high METTL1 level (TPM ≥ 23.442) and low METTL1 level (TPM < 23.442) in the TCGA cohort. Log-rank test. **c** ROC analysis for OS was conducted based on the METTL1 mRNA expression in the TCGA cohort. A cut-off value of 4.557 for METTL1 log_2_(TPM + 1) with a specificity of 0.695 and sensitivity of 0.700. **d** The protein levels of METTL1 in five representative paired PTC tumorous (T) tissues and adjacent normal tissues (N). **e** Representative images and semiquantitative analysis of immunohistochemistry (IHC) staining for METTL1 in a TMA with 48 cases of paired PTC tumorous and non-tumorous tissues. Chi-square test, ^***^P < 0.001. scale bars = 200 μm (50×). **f** Representative images of immunohistochemistry (IHC) staining for METTL1 protein in 48 cases of PTC tumorous tissues. Scale bar = 100 μm (100×) or 25 μm (400×). **g** Comparing the characteristics of lymph node metastasis, tumor diameter, AJCC stage, age, and gender between PTC patients with METTL1 high-expression (*n* = 27) and low-expression (*n* = 21). Chi-square test, the *P* value as indicated. **h**, **i** Univariate (**h**) and multivariate (**i**) Cox regression analyses for risk factors predicting OS outcome were performed in the TCGA cohort. The bars correspond to 95% confidence intervals. HR hazard ratio.

To explore the role of METTL1-mediated m^7^G tRNA modification in PTC, we first evaluated m^7^G tRNA methyltransferase METTL1 mRNA expression in PTCs from the GSE197443 dataset. Since METTL1 requires its cofactor WDR4 for its methyltransferase activity, we also analyzed WDR4 expression in the published dataset [40]. The expression of METTL1 and WDR4 in PTC tumor tissues was significantly greater than that in normal tissues (Figs. 1a and S1a). In addition, Kaplan–Meier survival analysis of the PTC transcriptome data from The Cancer Genome Atlas (TCGA) indicated that high METTL1 and WDR4 expression levels were strongly associated with shorter overall survival (OS) in patients with PTC (Figs. 1b and S1b). Moreover, receiver operating characteristic (ROC) curve analysis revealed that high METTL1 and WDR4 expression predicted worse OS in patients with PTC (Figs. 1c and S1c). To further confirm the above results obtained from public databases, we evaluated the expression of METTL1 in fresh frozen tumor tissues compared with that in matched adjacent normal thyroid tissues derived from patients with PTC and found that METTL1 expression was greater in PTC tumor tissues than in normal thyroid tissues (Figs. 1d and S1d). Moreover, we analyzed METTL1 and WDR4 protein expression in a PTC tissue microarray (TMA) containing 48 paired tumorous and adjacent normal thyroid tissues. The results revealed that METTL1 was upregulated in 56.3% of the PTC tumorous tissue samples but was upregulated in only 18.8% of the adjacent tissue samples (Fig. 1e). WDR4 was upregulated in 62.5% of the PTC tumorous tissue samples but only in 16.7% of the adjacent tissue samples (Fig. S1e). These data suggest that METTL1 and its co-factor WDR4 might play an oncogenic role in PTC. Next, we assessed the relationships between METTL1 or WDR4 expression levels and different clinicopathological characteristics in patients with PTC (Figs. 1f, g and S1f, g). Both METTL1 and WDR4 levels were positively correlated with lymph node metastasis in PTC patients (Figs. 1g and S1g). In addition, univariate and multivariate Cox regression analyses revealed that METTL1 expression was an independent predictor of the clinical outcomes of patients with PTC, with performance comparable to that of AJCC stage (Fig. 1h, i). Together, these results confirmed that the m^7^G tRNA methyltransferases METTL1 and WDR4 are pathologically and clinically associated with tumor aggressiveness and poor survival outcomes in patients with PTC.

### METTL1 promotes PTC cell proliferation and metastasis in a manner dependent on its tRNA methyltransferase activity

**Fig. 2:**
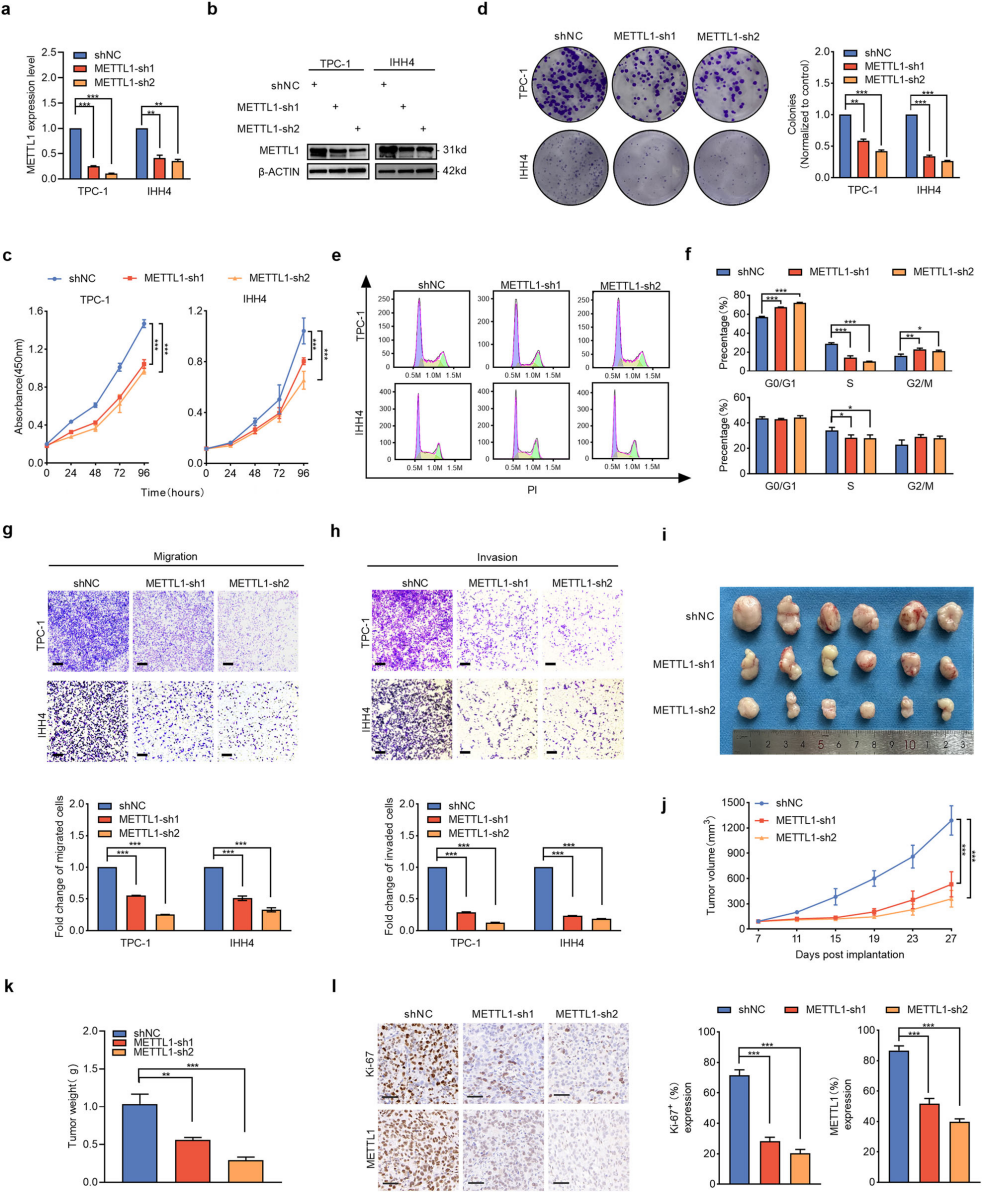
METTL1 promotes PTC cell proliferation and metastasis in a manner dependent on its tRNA methyltransferase activity. **a** RT-qPCR was performed to detect METTL1 mRNA expression in TPC-1 and IHH4 cells stably transfected with METTL1-shRNAs (METTL1-sh) or control shRNA (shNC) (*n* = 3). **b** Western blot was performed to detect METTL1 protein expression in TPC-1 and IHH4 cells stably transfected with METTL1-shRNAs (METTL1-sh) or control shRNA (shNC). **c** Cell viability assay by CCK-8 method to compare the proliferation rate between METTL1-depleted and control TPC-1 and IHH4 cells (*n* = 3). **d** Representative images and statistical bar graphs of the colony formation assays for TPC-1 and IHH4 cells with or without METTL1 knockdown (*n* = 3). **e**, **f** Cell cycle analysis and quantification of METTL1-depleted and control TPC-1 and IHH4 cells (*n* = 3). **g**, **h** Representative images (upper) and statistical bar graphs (lower) depicting the relative cell migration rate (**g**) and invasion rate (**h**) in TPC-1 and IHH4 cells with or without METTL1 deletion (*n* = 3). Scale bar = 200 μm **i**–**k** Representative images (**i**), tumor growth volume (**j**), and tumor weight (**k**) of subcutaneous xenograft tumors in METTL1-depleted and control K1 cells (*n* = 6). **l** Representative immunohistochemistry (IHC) staining images and semiquantitative analyses of Ki-67 (upper) and METTL1 (lower) of subcutaneous xenograft tumors in METTL1-depleted and control K1 cells. Scale bar = 50 μm. Two-tailed paired Student’s *t*-test in (**a**, **d**, **g**, and **h**), two-tailed unpaired Student’s *t*-test in (**f**, **k**, and **l**), One-way ANOVA test in (**c**, **j**) ^*^P < 0.05, ^**^P < 0.01, ^***^P < 0.001.

Given the hypothesis that oncogenic METTL1 drives the metastasis and poor survival outcomes of PTC patients, we assessed the biological function of METTL1 in PTC cells in vitro and in vivo. Compared with the normal thyroid cell line Nthy-ori 3-1, the PTC cell lines (TPC-1, IHH4, BCPAP, and K1) presented significantly greater METTL1 expression, while TPC-1 and IHH4 cells presented slightly higher METTL1 levels than BCPAP and K1 did (Fig. S2a). Therefore, TPC-1 and IHH4 cells were selected to establish METTL1-knockdown cell lines. METTL1 expression was silenced via the use of two independent shRNAs in PTC cell lines (Figs. 2a, b and S2b). The proliferation and colony formation ability of PTC cells were significantly reduced due to METTL1 loss (Fig. 2c, d). Cell cycle analysis revealed that METTL1 knockdown decreased the proportion of S-phase cells and that an increased proportion of G2/M-phase cells resulted in S-phase arrest of the cell cycle in PTC cells (Fig. 2e, f). These results suggested that knockdown of METTL1 blocked the cell cycle and thus inhibited PTC cell proliferation. In addition, we further explored the oncogenic effect of METTL1 on PTC metastasis. As shown in Fig. 2g, h, the invasive and migratory capacities of both PTC cell lines were markedly inhibited by METTL1 depletion. To further investigate the role of METTL1 in the development of malignant behaviors in PTC cells in vivo, we subcutaneously injected PTC cells into nude mice to generate a xenograft model in vivo. Nude mice injected with METTL1-knockdown cells had significantly slower tumor growth and lower tumor volume and weight than did those injected with parental control cells expressing METTL1 (Fig. 2i–k). IHC staining revealed that METTL1 expression was reduced in the METTL1-knockdown group, followed by decreased Ki-67 staining, indicating that METTL1 knockdown inhibited the proliferative activity of PTC cells in vivo (Fig. 2l). Next, to further confirm the effect of METTL1 on the lymph node metastasis of PTC cells in vivo, we established a nude mouse model of the lymph node metastasis of tumor cells injected into the footpad. Strikingly, METTL1 downregulation significantly reduced the volume of metastatic inguinal lymph node metastases (Fig. S2c, d). Taken together, these results indicate that METTL1 knockdown inhibits the proliferation and metastasis of PTC cells in vivo and in vitro.

**Fig. 3:**
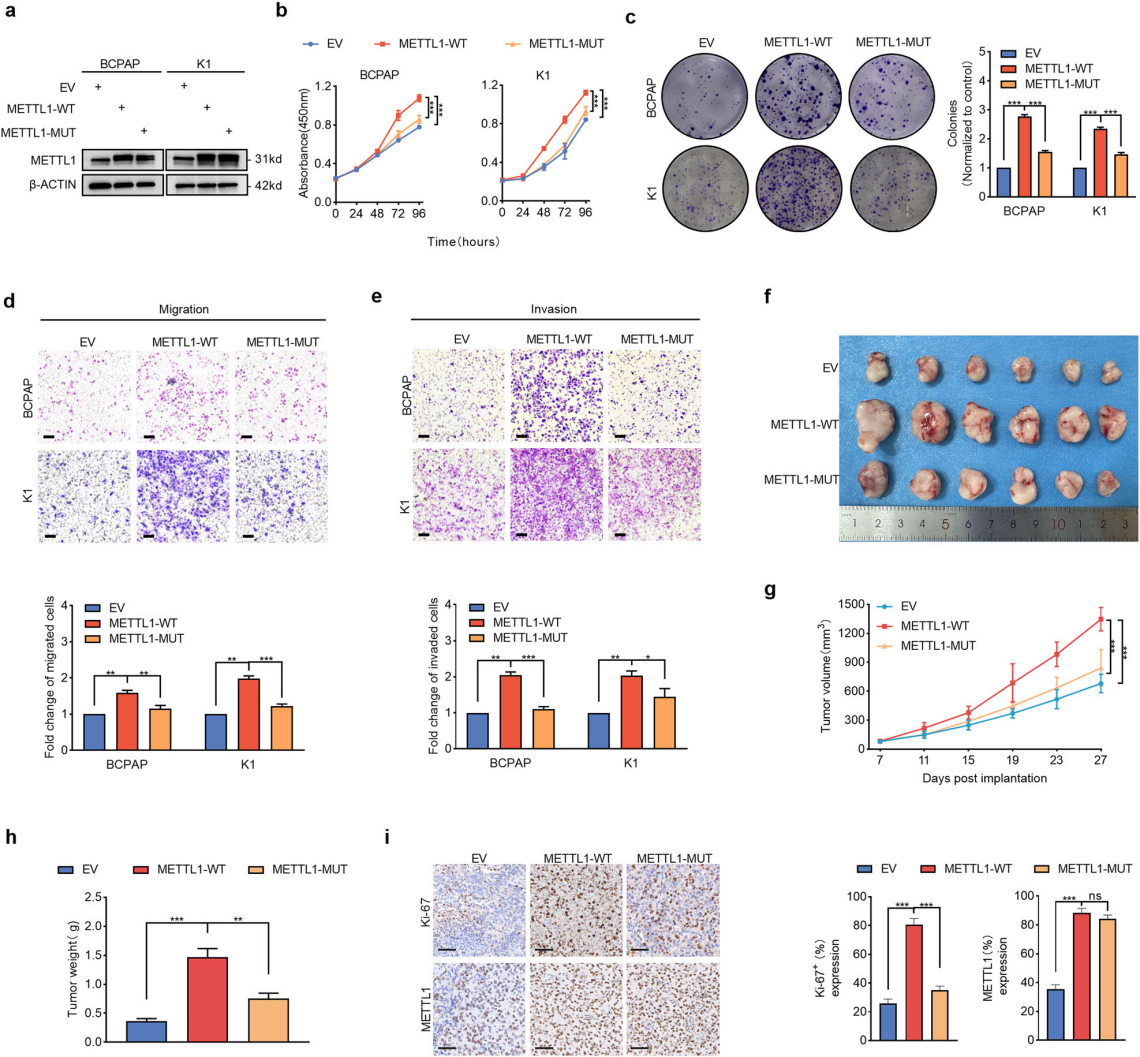
METTL1 promotes PTC cell proliferation and metastasis in a manner dependent on its tRNA methyltransferase activity. **a** Western blot was performed to detect METTL1 protein expression in BCPAP and K1 cells stably transfected with METTL1 wild-type construct (METTL1-WT), catalytic mutation construct (METTL1-MUT), or empty vector (EV). **b** Cell viability assay by CCK-8 method to compare the proliferation rate between METTL1-WT, METTL1-MUT, and EV stably expressed BCPAP and K1 cells (*n* = 3). **c** Representative images and statistical bar graphs of the colony formation assays in METTL1-WT, METTL1-MUT, or EV stably expressed BCPAP and K1 cells (*n* = 3). **d**, **e** Representative images (upper) and statistical bar graphs (lower) depicting the relative cell migration rate (**d**) and invasion rate (**e**) in METTL1-WT, METTL1-MUT, or EV stably expressed BCPAP and K1 cells (*n* = 3). Scale bar = 200 μm **f**–**h** Representative images (**f**), tumor growth volume (**g**), and tumor weight (**h**) of subcutaneous xenograft tumors in K1 cells stably transfected with METTL1-WT, METTL1-MU,T or EV. (*n* = 6). **i** Representative immunohistochemistry (IHC) staining images and semiquantitative analyses of Ki-67 (upper) and METTL1 (lower) of subcutaneous xenograft tumors in METTL1-WT, METTL1-MUT, or EV stably expressed K1 cells. Scale bar = 50 μm. Two-tailed paired Student’s *t*-test in (**c**–**e**), two-tailed unpaired Student’s *t*-test in (**h**, **i**), One-way ANOVA test in (**b**, **g**), ns non-significant, ^*^P < 0.05, ^**^P < 0.01, ^***^P < 0.001.

We then overexpressed METTL1 in both PTC cell lines to test whether the inhibitory effects on the proliferation and metastasis of PTC cells resulted from specific knockdown of METTL1 (Figs. 3a and S3a). The results showed that METTL1 overexpression promoted the proliferation, migration, and invasion of PTC cells (Fig. 3b–e). These data indicate that METTL1 specifically promotes PTC cell proliferation and metastasis.

The natural METTL1 protein possesses tRNA methyltransferase activity, but the loss-of-function mutation at leucine 160 and the aspartic acid 163 residue (L160/D163) of METTL1 inactivates its methyltransferase activity and reduces the stability of some tRNAs, such as tRNA-Arg-TCT [34]. To examine whether the methyltransferase activity of METTL1 is necessary for the proliferative and aggressive phenotype of PTC cells, we ectopically expressed a METTL1 catalytically disabled mutant construct (L160A/D163A), which lacks the ability to promote tRNA m^7^G modification in PTC cells (Fig. 3a). As shown in Fig. 3b–e, catalytically inactivated METTL1 did not increase the proliferative, invasive or migratory capacity of PTC cells. Consistent with the finding that METTL1 induces an accelerated proliferative and metastatic phenotype in PTC in vitro, the in vivo xenograft models indicated that the overexpression of the catalytically active wild-type METTL1 promoted the development of tumors in PTC cells, but the catalytically disabled mutant METTL1 failed to exert this effect (Fig. 3f–i). Collectively, these data indicate that METTL1 promotes the proliferation and metastasis of PTC cells in a manner dependent on its m^7^G tRNA methyltransferase activity.

### METTL1 regulates TNF-α expression at the posttranscriptional level

**Fig. 4:**
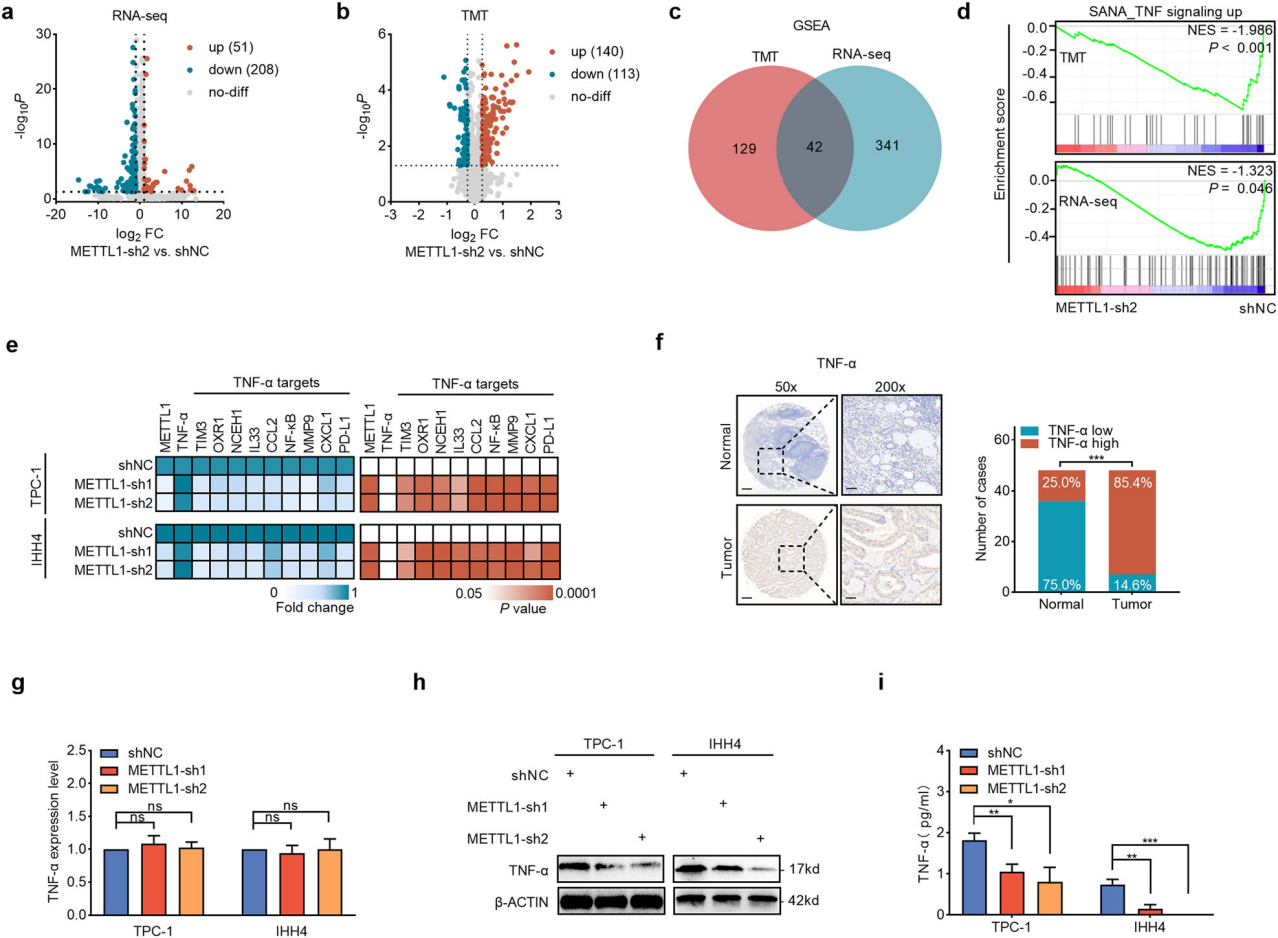
METTL1 regulates TNF-α expression at the posttranscriptional level. **a** The volcano plot of different expression genes (DEGs) between METTL1-depleted and control PTC cells by RNA-seq. **b** The volcano plot of different expression proteins (DEPs) between METTL1-depleted and control PTC cells by Tandem Mass Tag. **c** Venn diagram of DEPs-enriched GSEA pathways and DEGs-enriched GSEA pathways. **d** GSEA was conducted to show the enriched gene signatures in METTL1-control PTC cells. **e** RT-qPCR was performed to validate the representative differentially expressed TNF-α target genes in METTL1-depleted and control TPC-1 and IHH4 cells (*n* = 3). Unpaired Student’s *t*-test, the *P* value as indicated. **f** Representative images and semiquantitative analyses of immunohistochemistry (IHC) staining for TNF-α in a TMA with 48 cases of paired PTC tumorous and non-tumorous tissues. Chi-square test, ^***^P < 0.001. scale bars = 200 μm (50×) or 50 μm (200×). **g** RT-qPCR was performed to detect TNF-α mRNA expression in TPC-1 and IHH4 cells stably transfected with METTL1-shRNAs (METTL1-sh) or control shRNA (shNC) (*n* = 3). **h** Western blot was performed to detect TNF-α protein expression in TPC-1 and IHH4 cells stably transfected with METTL1-shRNAs (METTL1-sh) or control shRNA (shNC). **i** Enzyme-linked immunosorbent assay (ELISA) to compare the secretion between METTL1-depleted and control TPC-1 and IHH4 cells (*n* = 3). Two-tailed paired Student’s *t*-test in (**e**, **g**), two-tailed unpaired Student’s *t*-test in (**i**) ns non-significant, ^*^P < 0.05, ^**^P < 0.01, ^***^P < 0.001.

To identify the downstream targets regulated by METTL1, we performed transcriptome sequencing at the RNA level (RNA-seq) and tandem mass tag (TMT) experiments at the protein level in METTL1-knockdown and parental control PTC cells. A total of 208 transcriptionally downregulated genes and 113 posttranscriptionally downregulated proteins were screened in the METTL1-knockdown PTC cells (Fig. 4a, b). To screen the coaltered gene signatures via both transcriptional and posttranscriptional approaches, we performed GSEA and GO analysis of these dysregulated genes and proteins in METTL1-knockdown cells. The GSEA results revealed that a total of 42 overlapping signatures were altered in both the transcriptome and proteome, particularly in the TNF-α-mediated signaling pathway, which was significantly downregulated by METTL1 knockdown, with a higher rank in both the TMT and RNA-seq GSEA results (Figs. 4c, d and S4a). In addition, GO analysis revealed that METTL1 knockdown-mediated downregulation of genes and proteins was required for generating the biological processes of cell adhesion and population proliferation, which led to tumor proliferation and metastasis (Fig. S4b). TNF-α, a proinflammatory cytokine, is secreted by immune cells in the tumor microenvironment and is also secreted by tumor cells to play an oncogenic role in driving tumor progression and metastasis in multiple cancer types [41, 42], including PTC; in PTC, TNF-α induces epithelial‒mesenchymal transition to activate the metastatic and proliferative abilities of PTC cells [43]. Therefore, we assessed the expression of TNF-α and its downstream targets for further validation of their roles in METTL1-driven PTC progression and metastasis. As confirmed by real-time quantitative PCR (RT‒qPCR), the expression of TNF-α signaling downstream targets was significantly inhibited in METTL1-knockdown PTC cells (Fig. 4e). In addition, the TNF-α downstream target PI3K/AKT pathway was also inactivated in METTL1-knockdown PTC cells, with lower total protein levels of p110α, p85, and AKT and phosphorylated AKT protein expression (Fig. S4c). Moreover, we found that TNF-α is highly expressed in PTC tumor tissues compared with adjacent tissues, as assessed by immunohistochemistry (IHC) (Fig. 4f). These results suggest that TNF-α signaling might be a direct target in METTL1-mediated progression and metastasis of PTC.

To further reveal the regulatory relationship between METTL1 and TNF-α in PTC, we directly examined the expression and secretion of TNF-α in PTC cells with or without METTL1. Interestingly, METTL1 deletion did not alter TNF-α expression at the transcriptional level (Fig. 4g) but significantly decreased TNF-α protein levels (Figs. 4h and S4d) and secretion (Fig. 4i). Given these findings, we conclude that METTL1 regulates TNF-α expression at the posttranscriptional level to promote PTC progression and metastasis.

### METTL1-mediated m^7^G tRNA modification regulates the codon-specific translation of TNF-α

**Fig. 5:**
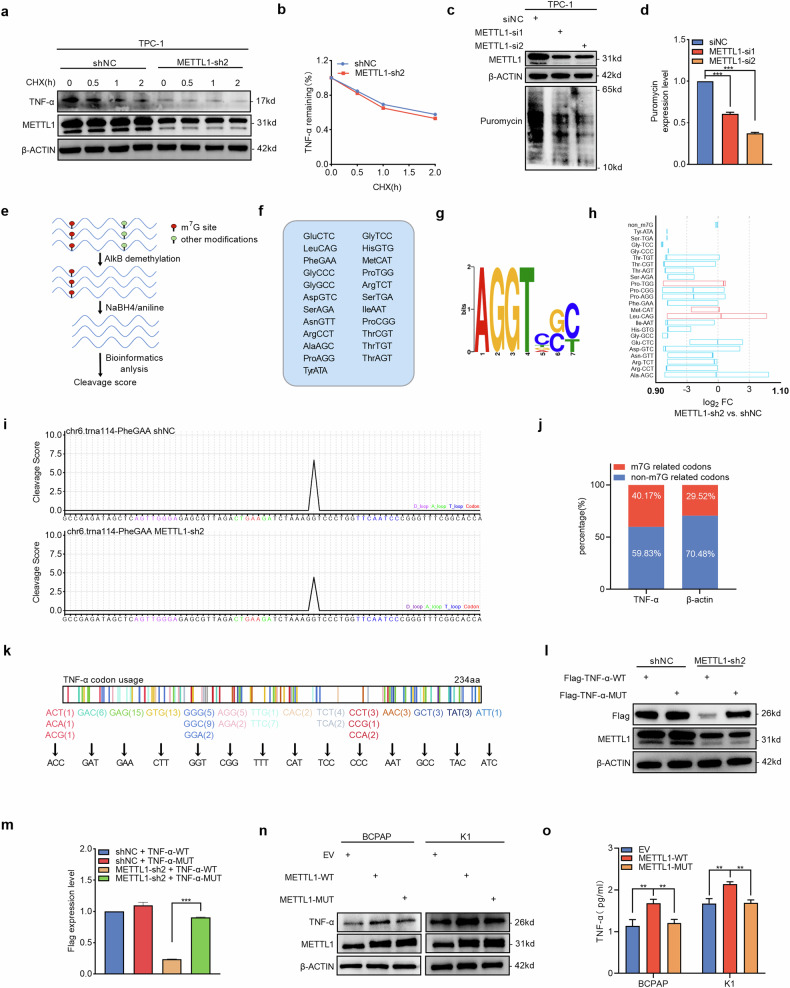
METTL1-mediated m7G tRNA modification regulates the codon-specific translation of TNF-α. **a**, **b** Western blot analysis of TNF-α and statistical chart of TNF-α relative expression level at the indicated time points after treatment with 100 µg/ml cycloheximide (CHX). **c**, **d** Puromycin intake assay of TPC-1 cells with or without METTL1 depletion using two independent siRNAs. Total protein samples were examined by western blot using anti-puromycin antibody (**c**). Quantitation of the bands is shown in (**d**). **e** Schematic diagram of the TRAC-seq method used to calculate cleavage scores. **f** List of 23 downregulated m^7^G-modified tRNAs identified by TRAC-seq in PTC cells. **g** Motif sequence at the m^7^G site was identified by TRAC-seq in PTC cells. **h** Expression profile of the 23 downregulated m^7^G-modified tRNAs. Each boxplot shows the expression of a tRNA type that was calculated from the combined expression of all tRNA genes for the same tRNA type. **i** Representative image showing the cleavage scores at the motif sequence from different groups. **j** Codon analysis of m^7^G tRNA decoded codons of TNF-α. **k** Schematic drawing of the synonymous mutations in m^7^G tRNA decoding codons in TNF-α cDNA sequence. **l**, **m** Western blot (**l**) and quantitation analysis (**m**) of protein expression of TNF-α in TPC-1 control and METTL1-depleted cells overexpressing TNF-α-WT-Flag, TNF-α mut-Flag. **n** Western blot was performed to detect TNF-α protein expression in METTL1-WT, METTL1-MUT, or EV stably expressed BCPAP and K1 cells. **o** ELISA to compare the secretion between METTL1-WT, METTL1-MUT, or EV stably expressed BCPAP and K1 cells (*n* = 3). Two-tailed paired Student’s *t*-test in (**b**, **d**, and **m**), two-tailed unpaired Student’s *t*-test in (**o**) ^**^P < 0.01, ^***^P < 0.001.

mRNA translation and protein degradation are two major processes involved in protein accumulation at the posttranscriptional level in cells. Given the hypothesis that METTL1 regulates TNF-α expression at the posttranscriptional level, we further investigated the exact posttranscriptional mechanism by which METTL1 specifically modulates TNF-α expression. The CHX-mediated protein degradation assay revealed that METTL1 did not alter TNF-α protein degradation in PTC cells (Fig. 5a, b), which suggested that the reduction in TNF-α protein expression in METTL1-knockdown cells does not result from protein stability but might be related to translation efficiency.

METTL1, as a methyltransferase, transfers methyl groups to the variable loop of tRNA to catalyze tRNA m^7^G modification. METTL1 can affect the m^7^G tRNA-mediated codon-specific translation of EGFR/EFEMP1 by regulating tRNA m^7^G modification to regulate bladder cancer cell behavior [44]. Thus, we speculate that METTL1 regulates TNF protein expression via m^7^G tRNA modification-mediated codon-specific translation in PTC cells. First, we evaluated whether translation impairment was caused by METTL1 knockdown in PTC cells by a puromycin intake assay. As shown in Fig. 5c, d, the incorporated puromycin significantly decreased in METTL1-knockdown PTC cells, which confirmed that METTL1 knockdown decreased global translation efficiency in PTC cells. Next, to explore whether the effect of METTL1 on translation efficiency depends on its catalytic activity in m^7^G tRNA modification, we performed m^7^G site-specific TRAC-seq to profile m^7^G modifications in tRNAs in PTC cells after METTL1 knockdown compared with those in parental cells (Fig. 5e). By TRAC-seq, we identified 23 tRNAs that were significantly downregulated in METTL1-knockdown PTC cells (Fig. 5f–h). METTL1 knockdown markedly reduced the m^7^G signals in the identified tRNAs (Fig. 5i). We subsequently performed codon frequency analysis, which revealed that these downregulated m^7^G tRNAs were more predominant among the TNF-α mRNAs than among the β-actin mRNAs (Fig. 5j). These data suggest that METTL1 catalyzes m^7^G modification in certain specific RNAs to regulate codon-specific translation of target proteins in PTC cells, thus binding more frequently to codons on TNF-α mRNAs and increasing translation efficiency.

To further explore whether TNF-α mRNA translation requires m^7^G tRNA modification, we generated cDNAs of TNF-α mutants in which m^7^G tRNA-decoded codons were replaced by their non-m^7^G tRNA-decoded synonymous counterparts (Fig. 5k). Equal amounts of wild-type or mutant TNF-α cDNA were transfected into METTL1-knockdown PTC cells and parental control cells. Western blotting analysis revealed that the wild-type TNF-α cDNA constructs were not efficiently expressed in METTL1-knockdown cells, but the mutant TNF-α cDNA constructs with m^7^G tRNA modification-independent codons were translated at almost equivalent levels in METTL1-knockdown cells as in parental control cells (Fig. 5l, m), which suggested that METTL1 regulates TNF-α mRNA translation via the m^7^G tRNA modification-mediated codon-specific translation approach. Next, we examined TNF-α protein expression and secretion in PTC cells ectopically expressing wild-type METTL1 constructs or catalytically dead mutant METTL1 constructs. As shown in Figs. 5n, o and S5a. The overexpression of wild-type METTL1 promotes the protein expression and secretion of TNF-α in PTC cells, but catalytically dead mutant METTL1 fails, which suggests that METTL1-mediated regulation of TNF-α expression is dependent on its methyltransferase activity. Taken together, these results confirm that METTL1-mediated m^7^G tRNA modifications regulate the codon-specific translation of TNF-α.

### TNF-α is essential for METTL1-driven progression and metastasis

**Fig. 6:**
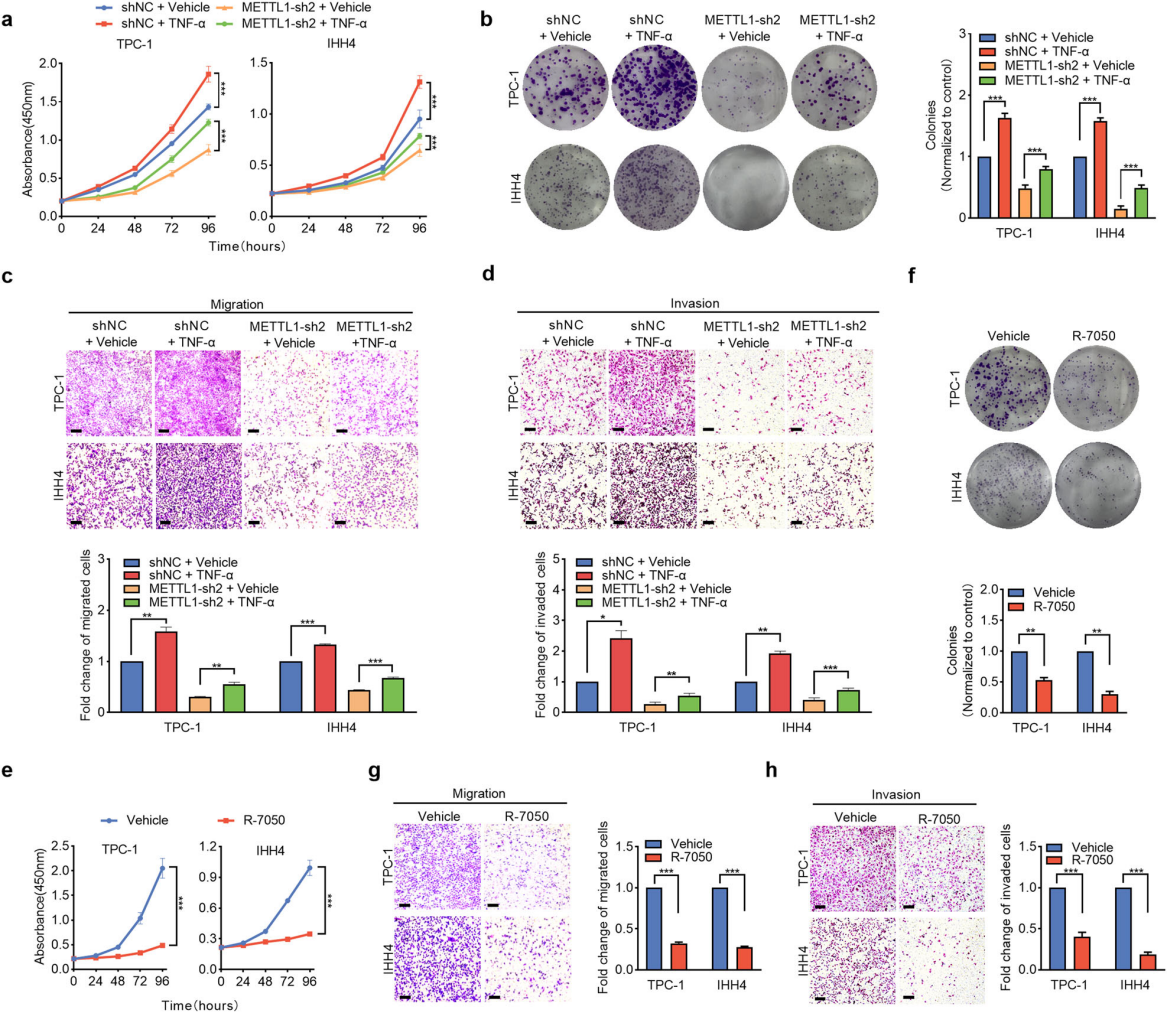
TNF-α is essential for METTL1-driven progression and metastasis. **a** METTL1-depleted and control TPC-1 and IHH4 cells were stimulated with 50 ng/ml exogenous TNF-α recombinant protein (r-TNF-α) and vehicle for 48 h, and then compared for cellular viability by the CCK8 method (*n* = 3). **b** Representative images and statistical bar graphs of the colony formation assays for METTL1-depleted and control TPC-1 and IHH4 cells with or without stimulation of 50 ng/ml exogenous r-TNF-α for 48 h (*n* = 3). **c**, **d** Representative images (upper) and statistical bar graphs (lower) depicting the relative cell migration rate (**c**) and invasion rate (**d**) in METTL1-depleted and control TPC-1 and IHH4 cells with or without stimulation of 50 ng/ml exogenous r-TNF-α for 48 h (*n* = 3). Scale bar = 200 μm. **e** Cell viability assay by CCK-8 method to compare the proliferation rate between wild-type TPC-1 and IHH4 cells with or without stimulation of 1.5 μM R-7050 for 6 h (*n* = 3). **f** Representative images and statistical bar graphs of the colony formation assays for wild-type TPC-1 and IHH4 cells with or without stimulation of 1.5 μM R-7050 for 6 h. (*n* = 3). **g**, **h** Representative images (left) and statistical bar graphs (right) depicting the relative cell migration rate (**g**) and invasion rate (**h**) in wild-type TPC-1 and IHH4 cells with or without stimulation of 1.5 μM R-7050 for 6 h (*n* = 3). Scale bar = 200 μm. Two-tailed paired Student’s *t*-test in (**b**–**d** and **f**–**h**), One-way ANOVA test in (**a**, **e**) ^*^P < 0.05, ^**^P < 0.01, ^***^P < 0.001.

To verify the essential role of TNF-α in METTL1-driven accelerated proliferation and enhanced metastasis of PTC cells, we further performed a rescue assay by utilizing recombinant TNF-α protein in METTL1-knockdown and control cells. We found that supplementation with TNF-α recombinant protein partially restored the proliferative ability of PTC cells both in vitro and in vivo (Figs. 6a, b and S6a–d), which was inhibited by METTL1 loss in METTL1-knockdown PTC cells. Similar findings in terms of metastatic ability were observed in TNF-α recombinant protein-rescued METTL1-knockdown cells (Fig. 6c, d). In addition, we used an inhibitor of TNF-α, R-7050, which is a class of triazoloquinoxalines that selectively inhibits TNF-α-induced cell signaling [45]. As shown in Fig. 6e–h, the TNF-α inhibitor R-7050 specifically inhibited the proliferation (Fig. 6e, f) and metastasis (Fig. 6g, h) of wild-type PTC cells. Overall, these data revealed that TNF-α is a predominant downstream target and is required for METTL1-mediated proliferation and metastasis of PTC.

### Clinical relevance of METTL1, WDR4, and TNF-α in PTC

**Fig. 7:**
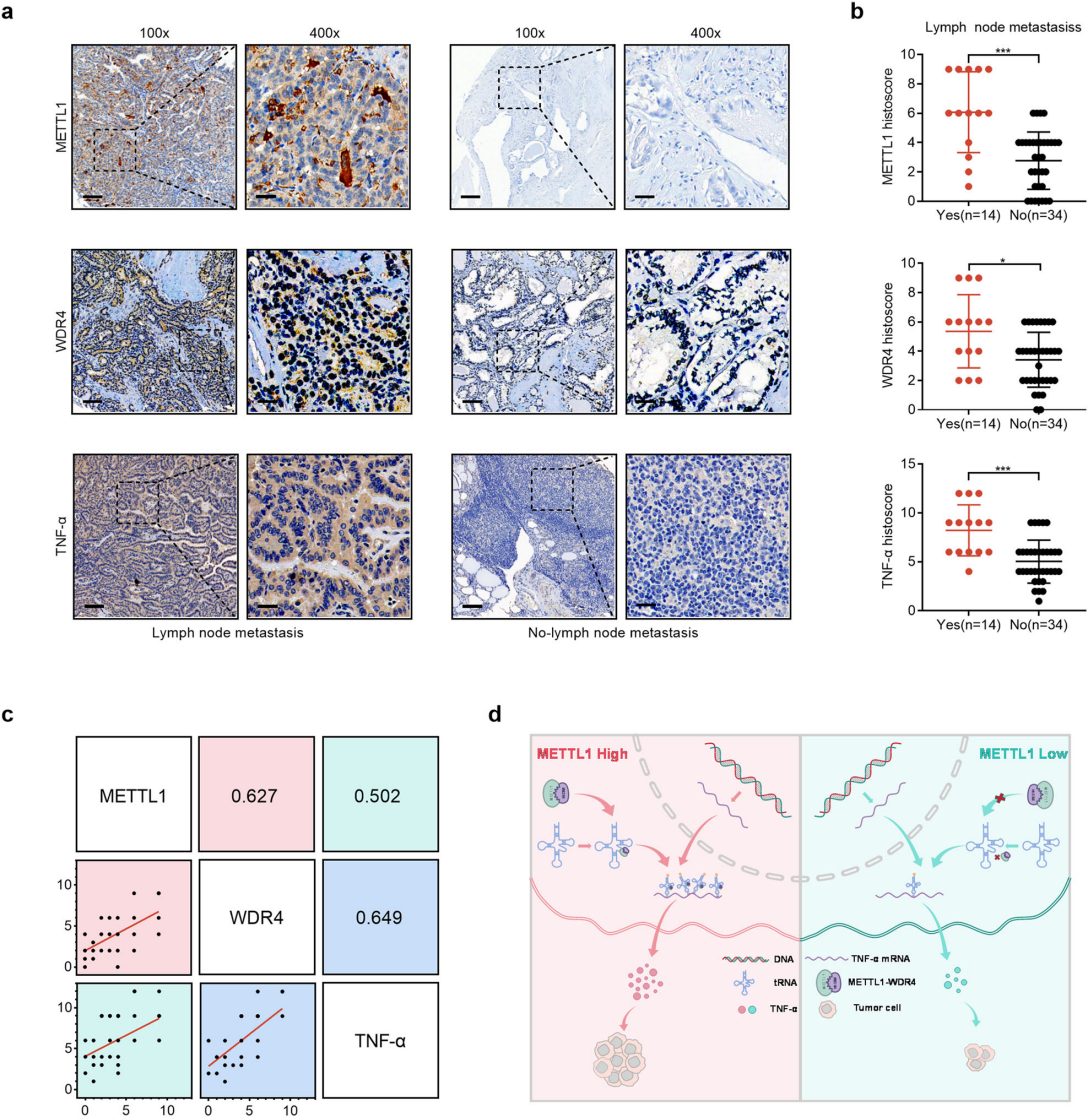
Clinical relevance of METTL1, WDR4, and TNF-α in PTC. **a** Representative immunohistochemistry of METTL1 (upper), WDR4 (middle), and TNF-α (lower) proteins in PTC tissues from patients with (*n* = 14) or without (*n* = 34) lymph node metastasis. Scale bar = 100 μm (100×) or 25 μm (400×). **b** Statistical analysis of the histoscore of METTL1 (upper), WDR4 (middle), and TNF-α (lower) proteins in PTC tissues with (*n* = 14) or without (*n* = 34) lymph node metastasis. Mann–Whitney *U*-test, ^*^P < 0.05, ^***^P < 0.001. **c** Correlations among METTL1, WDR4 and TNF-α levels in PTC tissues (*n* = 48). Pearson correlation coefficient test. **d** Schematic diagram for METTL1 cooperates with WDR4 to drive the proliferation and metastasis of PTC through regulating the translation of TNF-α translation.

To investigate the clinical significance of METTL1-mediated codon-specific translation of TNF-α in PTC patients, we utilized PTC tumor tissues to validate the translational and clinical relevance of METTL1, WDR4, and TNF-α interactions. By analyzing the protein expression of METTL1, WDR4, and TNF-α in PTC tumor tissues, we found that PTC tissues from patients with lymph node metastasis had higher METTL1, WDR4, and TNF-α expression than those from patients without lymph node metastasis did (Fig. 7a, b). In addition, the expression level of METTL1 was positively correlated with that of WDR4 and TNF-α in PTC tumor tissues (Fig. 7c), and WDR4 expression was also positively correlated with TNF-α expression (Fig. 7c).

Taken together, our findings showed that the m^7^G tRNA methyltransferase METTL1 specifically catalyzes m^7^G tRNA modification to regulate codon-specific translation-mediated TNF-α translation to drive the proliferation and metastasis of PTC cells (Fig. 7d).

## Discussion

The correlation between mRNA abundance and protein expression in cancer is low, and proteins are expressed at a thousand-fold higher level than mRNA transcripts. In addition, cancer cells selectively promote the translation of oncogenes; thus, the expression of oncoproteins is greater than that of oncogene transcripts and facilitates cancer progression and metastasis [46, 47]. Therefore, targeting this selective translation may lead to promising cancer treatment strategies. As a bridge between mRNA and protein expression, tRNA is a key tool in the translation of mRNA into proteins and is heavily modified [9]. Many reports point to a clear link between defective tRNA modifications and human diseases such as type 2 diabetes and cancer [33]. However, the role and mechanisms of tRNA modifications in gene expression regulation and cancer progression are still poorly understood. Among them, METTL1, as a methyltransferase, mediates the m^7^G modification of tRNAs and plays an important role in regulating a variety of biological functions, including yeast growth under heat stress [48], self-renewal and differentiation of embryonic stem cells [30], and even the occurrence of various tumors, including digestive tumors, urological tumors, and hematologic tumors [16, 44, 49–51]; however, its potential role in PTC, including tumor progression and metastasis, is still largely unclear.

Here, we found that the expression of the m^7^G tRNA methyltransferase METTL1 is low in normal tissues but significantly upregulated in PTC tumor samples, and that high expression of METTL1 is associated with poor patient survival outcomes. Functionally, METTL1 depletion significantly inhibited the proliferative and metastatic activity of PTC cells and inhibited the growth of PTCs in a xenograft mouse model in vivo. These findings provide strong direct evidence for an important oncogenic role of METTL1 in PTC initiation and progression. The enzymatic activity of METTL1, a methyltransferase, is key to its role in diseases such as cancer. It has been reported that the L160A, D163A synonymous mutation located in the peptide chain of METTL1 is a loss-of-function mutation that impairs the methyltransferase activity of METTL1 [30]. Upon mutation of these active sites, the pro-proliferative and metastatic effects produced by METTL1 in PTC are greatly reduced. These findings strongly confirm that METTL1 drives PTC cell proliferation and metastasis through the catalytic activity of its methyltransferase.

Mechanistically, we used an antibody-independent TRAC-seq approach previously developed by Lin et al. [38] and revealed that deletion of METTL1 results in reduced expression of certain m^7^G-modified tRNAs and reduces mRNA translation in an m^7^G-related codon-dependent manner. Interestingly, another study revealed that METTL1 promotes cancer metastasis by catalyzing the upregulation of specific tRNA types by m^7^G, thereby promoting the translation of certain cancer-related genes [52]. Consistent with our data, these observations reveal the importance of tRNA recognition of codons regulating translation as a step in cancer. Indeed, the molecular mechanism of METTL1 is highly complex and multifaceted. The downstream molecules it regulates by modulating the translation process are also diverse. It has been reported to play an oncogenic role in colon cancer through the HMGA2 axis and to promote hepatocellular carcinoma through the PTEN pathway [53]. Thus, we sought to identify potential targets of METTL1 in PTC via multiomics (transcriptome, proteome, and m^7^G tRNA methylome) analysis. The results showed that METTL1-mediated modification of m^7^G tRNA upregulated TNF-α expression. These analyses provide evidence that METTL1-mediated m^7^G tRNA modifications regulate the codon-specific translation of TNF-α.

TNF-α, an inflammatory cytokine, is considered an anticancer substance in the early stage that promotes the necrosis of transplanted tumors in mice [54–56], but with increasing research, it was found to have an oncogenic effect completely opposite to that reported in previous studies [57]. For example, in prostate cancer, TNF-α levels are positively correlated with disease severity [58] and promote the growth and spread of various cancer cells, including those in the skin, ovary, pancreas, pleural cavity, and intestinal tract [59–63]. However, the role of TNF-α in PTC has never been reported. In this study, we found that METTL1 promotes PTC progression and metastasis by activating TNF-α signaling through the regulation of TNF-α translation. More importantly, the addition of exogenous TNF-α reversed the inhibitory effect of METTL1 deficiency on the proliferation and metastasis of PTC cells in vitro. Overall, we comprehensively analyzed the relationships among tRNA abundance, codon usage, and TNF-α translation, revealing the molecular mechanisms underlying the oncogenic function of METTL1 in PTC.

In this study, GO analysis revealed that the PI3K/AKT pathway was altered in METTL1-knockdown PTC cells. Some studies have shown that TNF-α directly or indirectly regulates the PI3K/AKT pathway. On the one hand, TNF-α binds to TNFR2 and increases the expression of tumor necrosis factor receptor-associated factor (TRAF) to bind and activate PI3K, which mediates AKT phosphorylation, thereby promoting cell proliferation [64–66]. On the other hand, TNF-α promotes the expression of the PI3K protein by activating the transcription factor NF-κB, thereby promoting cell proliferation [67, 68]. Therefore, we speculate that METTL1 may regulate PI3K/AKT signaling pathway activity by inducing TNF-α translation and activating TNF-α signaling. However, the role of the PI3K/AKT signaling pathway in the METTL1-driven progression and metastasis of PTC remains to be further explored.

Metastasis is a crucial factor affecting the prognosis of patients with PTC, but its mechanism is poorly understood [69]. By analyzing the clinical characteristics of PTC patients, we found that METTL1/WDR4, together with TNF-α, drive metastasis in PTC. These findings suggest that METTL1 is a highly promising new target for overcoming PTC metastasis. In recent years, many inhibitors that target RNA modifications, such as small-molecule inhibitors of FTO and ALKBH5, which inhibit immune escape from tumors by targeting m6A modifications to inhibit the growth of many cancers, have been developed and investigated in clinical studies [70, 71]. However, inhibitors against m^7^G modifications have not been found to be effective, so the targeting of METTL1 for tumor treatment is not yet feasible, and our study is slightly limited in terms of clinical translation.

In conclusion, the present study revealed that METTL1/WDR4 expression and m^7^G tRNA modification have powerful physiological functions in the regulation of TNF-α mRNA translation and PTC progression and metastasis. Our data reveal the mechanism by which METTL1 mediates PTC development and provide a molecular basis for the establishment of new therapeutic strategies for PTC.

## Supplementary information

Supplementary

Full and uncropped western blots

## Data Availability

The data and materials that support the findings of this study are available from the corresponding author upon reasonable request.
