# Supplementary material for: METTL1-mediated m7G tRNA modification drives papillary thyroid cancer progression and metastasis by regulating the codon-specific translation of TNF-α
Source: Cell Death Dis. 2025 May 14;16(1):378. doi: 10.1038/s41419-025-07716-8 (PMC12075834; doi:10.1038/s41419-025-07716-8)
Supplement: Supplementary file 1 — Supplementary [file 41419_2025_7716_MOESM1_ESM.pdf]

Supplementary material for

METTL1-mediated m7G tRNA modification drives papillary thyroid cancer progression and metastasis by regulating codon-specific translation of TNF-α

Weiwei Li, et al.

Contents:

6 Supplementary Figures and Legends

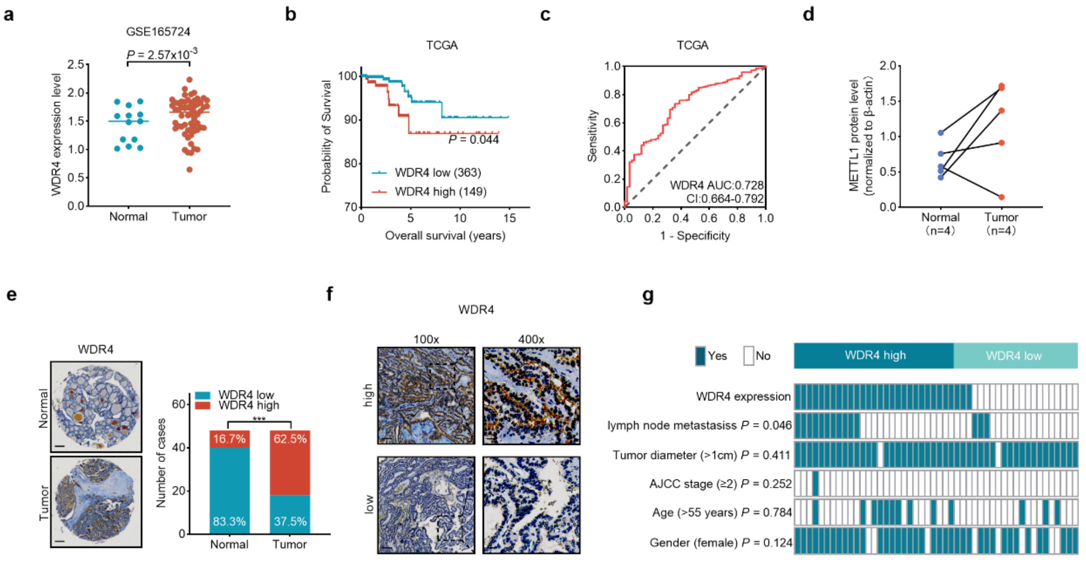

Fig. S1. METTL1 expression is high in PTC, and this phenotype is associated with a poor prognosis, related to Fig. 1. a) WDR4 mRNA levels in PTC tumorous tissues and adjacent normal tissues in a GEO dataset, GSE165724. b) Overall survival (OS) was compared between PTC patients with high WDR4 level (TPM ≥ 11.329) and low WDR4 level (TPM < 11.329) in TCGA cohort. Log-rank test. c) Receiver operating characteristic (ROC) analysis for overall survivals was conducted based on the WDR4 mRNA expression in TCGA cohort. A cut-off value of 3.647 for WDR4 $\log_2$(TPM+1) with a specificity of 0.644 and sensitivity of 0.734. d) Statistical analysis of METTL1 protein levels in 5 representative paired PTC tumorous tissues and adjacent normal tissues, related to Figure 1d. e) Representative images and semiquantitative analysis of immunohistochemistry (IHC) staining for WDR4 in a tissue microarray (TMA) with 48 cases of paired PTC tumorous and non- tumorous tissues. Chi-square test, ****P* < 0.001. scale bars = 200μm (50 ×). f) Representative images of immunohistochemistry (IHC) staining for WDR4 protein in 48 cases of PTC tumorous tissues. Scale bar = 100μm (100 ×) or 25μm (400 ×). g) Comparing the characteristics of lymph node metastasis, tumor diameter, AJCC stage, age, and gender between PTC patients with WDR4 high-expression (n = 30) and low-expression (n = 18). Chi-square test, the *P* value as indicated.

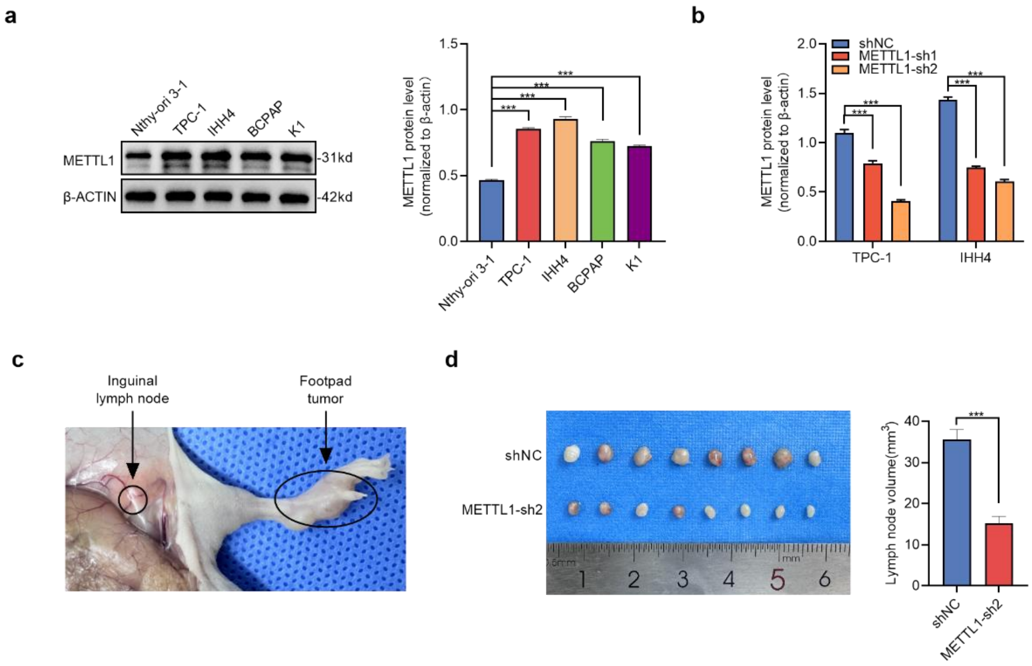

Fig. S2. METTL1 promotes PTC cell proliferation and metastasis in a manner dependent on its tRNA methyltransferase activity, related to Fig. 2. a) Western blot and quantitation analysis to compare METTL1 expression in normal thyroid cells (Nthy-ori 3-1) and PTC cells (TPC-1, IHH4, BCPAP and K1). b) Statistical analysis of METTL1 protein levels in TPC-1 and IHH4 cells stably transfected with METTL1-shRNAs (METTL1-sh) or control shRNA (shNC), related to Fig. 2b. c) An inguinal lymph node metastasis model was established by inoculating the foot pads of nude mice with K1 cells. d) Representative image and quantification of the volume of the inguinal lymph nodes (n = 8). Two tailed unpaired student's t-tests in (a, b, d), ***P<0.001.

a

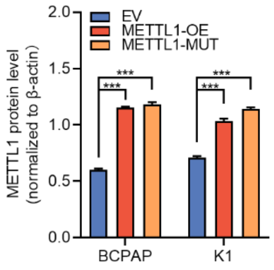

Fig. S3. METTL1 promotes PTC cell proliferation and metastasis in a manner dependent on its tRNA methyltransferase activity, related to Fig. 3. a) Statistical analysis of METTL1 protein levels in BCPAP and K1 cells stably transfected with METTL1 wild-type construct (METTL1-WT), catalytic mutation construct (METTL1-MUT), or empty vector (EV), related to Fig. 3a. Two tailed unpaired Student's t test, ***P<0.001.

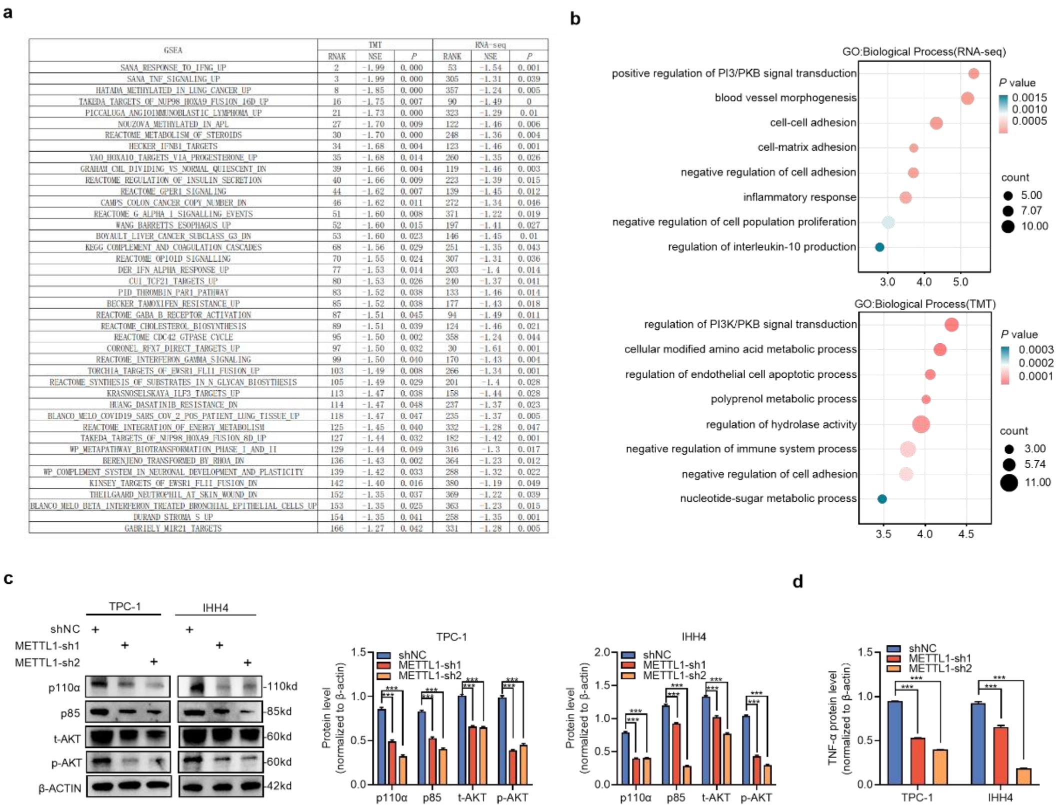

GSEA	TMT			RNA-seq		
	RNAK	NSE	*P*	RANK	NSE	*P*
SANA_RESPONSE_TO_IFNG_UP	2	-1.99	0.000	53	-1.54	0.001
SANA_TNF_SIGNALING_UP	3	-1.99	0.000	305	-1.31	0.039
HATADA_METHYLATED_IN_LUNG_CANCER_UP	8	-1.85	0.000	357	-1.24	0.005
TAKEDA_TARGETS_OF_NUP98_HOXA9_FUSION_16D_UP	16	-1.75	0.007	90	-1.49	0
PICCALUGA_ANGIOIMMUNOBLASTIC_LYMPHOMA_UP	21	-1.73	0.000	323	-1.29	0.01
NOUZOVA_METHYLATED_IN_APL	27	-1.70	0.009	122	-1.46	0.006
REACTOME_METABOLISM_OF_STEROIDS	30	-1.70	0.000	248	-1.36	0.004
HECKER_IFNB1_TARGETS	34	-1.68	0.004	123	-1.46	0.001
YAO_HOXA10_TARGETS_VIA_PROGESTERONE_UP	35	-1.68	0.014	260	-1.35	0.026
GRAHAM_CML_DIVIDING_VS_NORMAL_QUIESCENT_DN	39	-1.66	0.004	119	-1.46	0.003
REACTOME_REGULATION_OF_INSULIN_SECRETION	40	-1.66	0.009	223	-1.39	0.015
REACTOME_GPER1_SIGNALING	44	-1.62	0.007	139	-1.45	0.012
CAMPS_COLON_CANCER_COPY_NUMBER_DN	46	-1.62	0.011	272	-1.34	0.046
REACTOME_G_ALPHA_I_SIGNALLING_EVENTS	51	-1.60	0.008	371	-1.22	0.019
WANG_BARRETTS_ESOPHAGUS_UP	52	-1.60	0.015	197	-1.41	0.027
BOYAULT_LIVER_CANCER_SUBCLASS_G3_DN	53	-1.60	0.023	146	-1.45	0.01
KEGG_COMPLEMENT_AND_COAGULATION_CASCADES	68	-1.56	0.029	251	-1.35	0.043
REACTOME_OPIOID_SIGNALLING	70	-1.55	0.024	307	-1.31	0.036
DER_IFN_ALPHA_RESPONSE_UP	77	-1.53	0.014	203	-1.4	0.014
CUI_TCF21_TARGETS_UP	80	-1.53	0.026	240	-1.37	0.041
PID_THROMBIN_PAR1_PATHWAY	83	-1.52	0.038	133	-1.46	0.014
BECKER_TAMOXIFEN_RESISTANCE_UP	85	-1.52	0.038	177	-1.43	0.018
REACTOME_GABA_B_RECEPTOR_ACTIVATION	87	-1.51	0.045	94	-1.49	0.011
REACTOME_CHOLESTEROL_BIOSYNTHESIS	89	-1.51	0.039	124	-1.46	0.021
REACTOME_CDC42_GTPASE_CYCLE	95	-1.50	0.002	358	-1.24	0.044
CORONEL_RFX7_DIRECT_TARGETS_UP	97	-1.50	0.032	30	-1.61	0.001
REACTOME_INTERFERON_GAMMA_SIGNALING	99	-1.50	0.040	170	-1.43	0.004
TORCHIA_TARGETS_OF_EWSR1_FLI1_FUSION_UP	103	-1.49	0.008	266	-1.34	0.001
REACTOME_SYNTHESIS_OF_SUBSTRATES_IN_N_GLYCAN_BIOSYTHESIS	105	-1.49	0.029	201	-1.4	0.028
KRASNOSELSKAYA_ILF3_TARGETS_UP	113	-1.47	0.038	158	-1.44	0.028
HUANG_DASATINIB_RESISTANCE_DN	114	-1.47	0.048	237	-1.37	0.023
BLANCO_MELO_COVID19_SARS_COV_2_POS_PATIENT_LUNG_TISSUE_UP	118	-1.47	0.047	235	-1.37	0.005
REACTOME_INTEGRATION_OF_ENERGY_METABOLISM	125	-1.45	0.040	332	-1.28	0.047
TAKEDA_TARGETS_OF_NUP98_HOXA9_FUSION_8D_UP	127	-1.44	0.032	182	-1.42	0.001
WP_METAPATHWAY_BIOTRANSFORMATION_PHASE_I_AND_II	129	-1.44	0.049	316	-1.3	0.017
BERENJENO_TRANSFORMED_BY_RHOA_DN	136	-1.43	0.002	364	-1.23	0.012
WP_COMPLEMENT_SYSTEM_IN_NEURONAL_DEVELOPMENT_AND_PLASTICITY	139	-1.42	0.033	288	-1.32	0.022
KINSEY_TARGETS_OF_EWSR1_FLII_FUSION_DN	142	-1.40	0.016	380	-1.19	0.049
THEILGAARD_NEUTROPHIL_AT_SKIN_WOUND_DN	152	-1.35	0.037	369	-1.22	0.039
BLANCO_MELO_BETA_INTERFERON_TREATED_BRONCHIAL_EPITHELIAL_CELLS_UP	153	-1.35	0.025	363	-1.23	0.015
DURAND_STROMA_S_UP	154	-1.35	0.041	258	-1.35	0.001
GABRIELY_MIR21_TARGETS	166	-1.27	0.042	331	-1.28	0.005

Fig. S4. METTL1 regulates TNF-α expression at the posttranscriptional level, related to Fig. 4. a) Table listing the 42 overlapped pathways identified by GSEA between Tandem Mass Tags (TMT) and RNA sequencing (RNA-seq) results. b) Gene Ontology (GO) analysis was conducted to show the enriched biological process of RNA-seq (upper) and TMT (lower). c) Western blot and statistical analysis of the total p110α, p85, AKT protein and phosphorylated AKT protein. d) Statistical analysis of TNF-α protein levels in TPC-1 and IHH4 cells stably transfected with METTL1-shRNAs (METTL1-sh) or control shRNA (shNC), related to Fig. 4i. Two tailed unpaired Student's t test in (c, d), ***P<0.001.

a

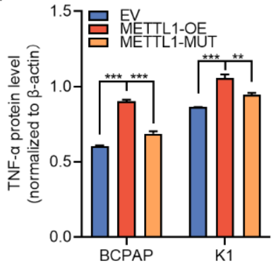

Fig. S5. METTL1-mediated m7G tRNA modification regulates the codon-specific translation of TNF-α, related to Fig. 5. a) Statistical analysis of TNF-α protein levels in BCPAP and K1 cells stably transfected with METTL1 wild-type construct (METTL1-WT), catalytic mutation construct (METTL1-MUT), or empty vector (EV)., related to Fig. 5n. Two tailed unpaired Student's t test, **P<0.01, ***P<0.001.

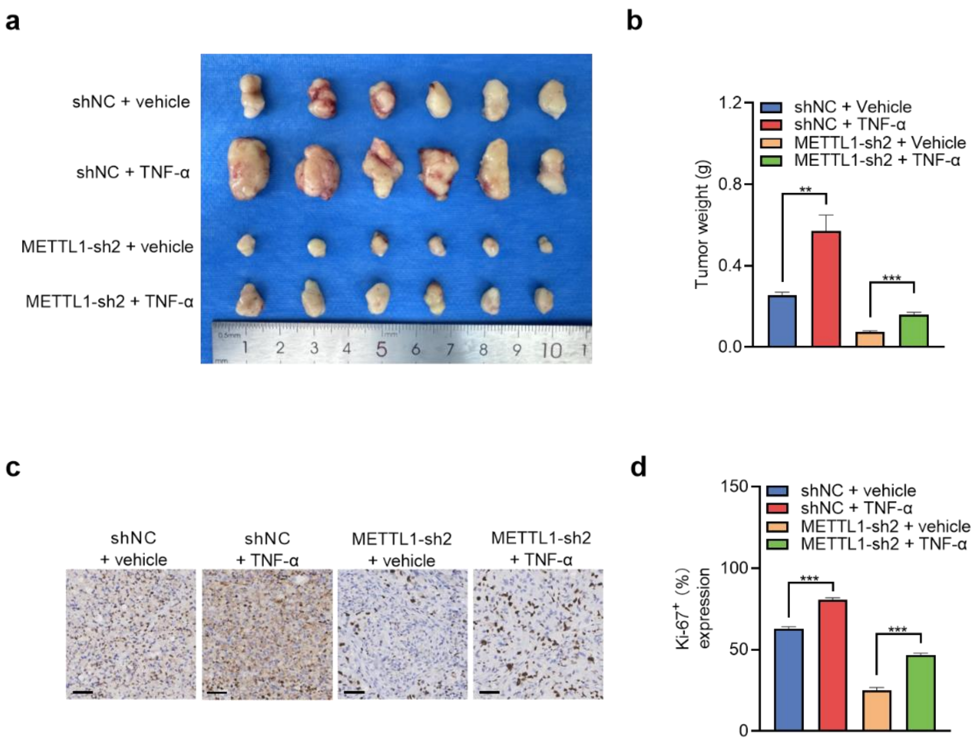

Fig. S6. TNF-α is essential for METTL1-driven progression and metastasis, related to Fig. 6. a) Subcutaneous xenografts of nude mice treated with vehicle and Recombinant TNF-α (50 μg/kg) in METTL1-knockdown and control K1 cells (n = 6). b) Tumor weight of xenograft tumors. c, d) Representative immunohistochemistry (IHC) staining images (c) and semiquantitative analyses (d) of Ki-67 of subcutaneous xenograft of nude mice treated with vehicle and Recombinant TNF-α (50 μg/kg) in METTL1-knockdown and control K1 cells. Scale bar = 50μm. Two tailed unpaired Student's t test in (b, d), ***P*<0.01, ****P*<0.001.
