# Supplementary material for: METTL1-mediated m7G tRNA modification drives papillary thyroid cancer progression and metastasis by regulating the codon-specific translation of TNF-α
Source: Cell Death Dis. 2025 May 14;16(1):378. doi: 10.1038/s41419-025-07716-8 (PMC12075834; doi:10.1038/s41419-025-07716-8)
Supplement: Supplementary file 2 — Full and uncropped western blots [file 41419_2025_7716_MOESM2_ESM.pdf]

**Figure 1d**

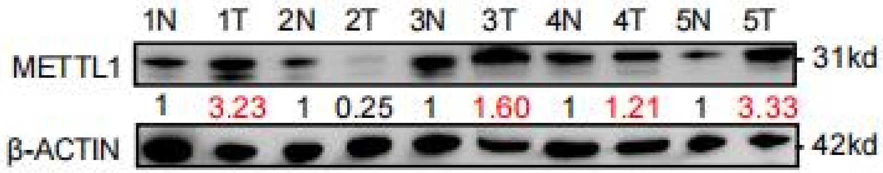


**METTL1**

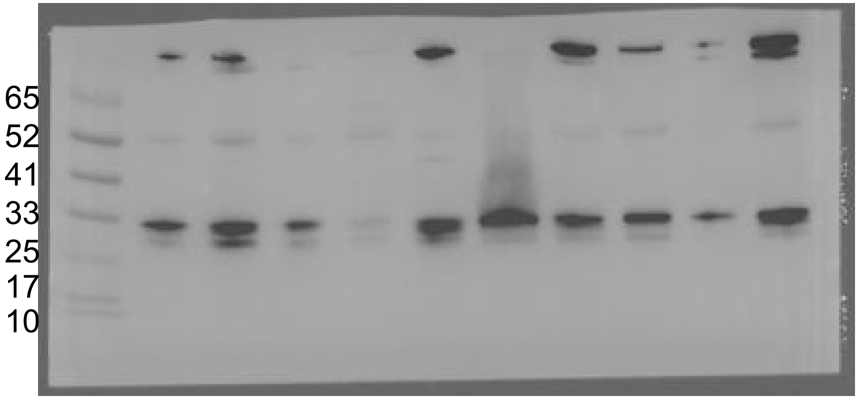


**β-ACTIN**

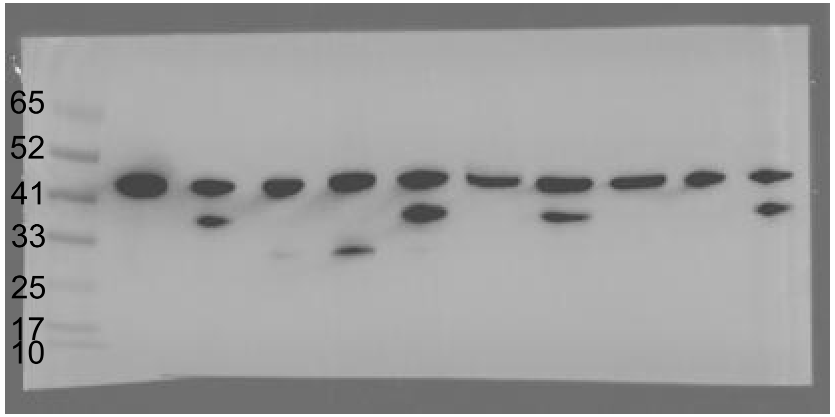

## Figure 2b

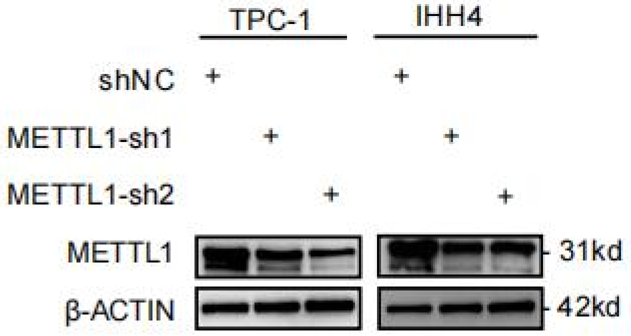


**METTL1**

TPC-1

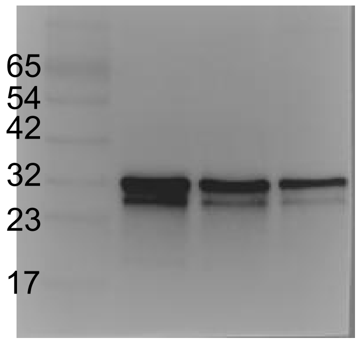


IHH4

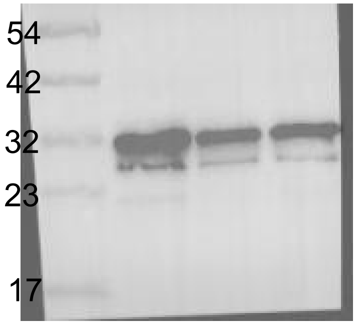


**β-ACTIN**

TPC-1

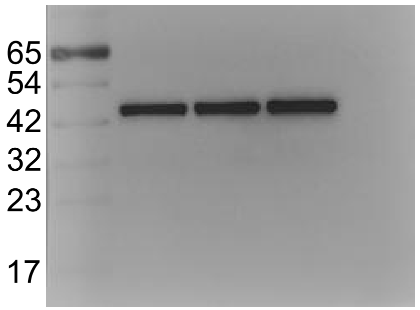


IHH4

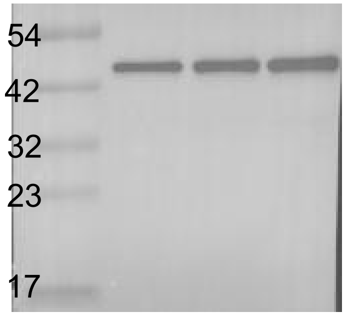

## Figure 3a

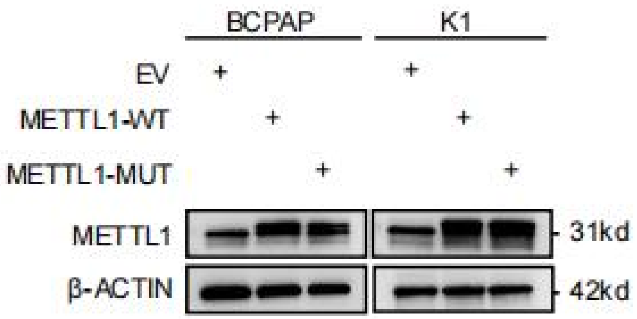


**METTL1**

BCPAP

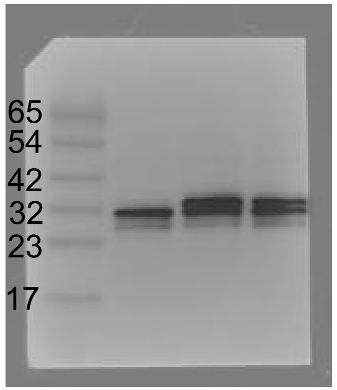


K1

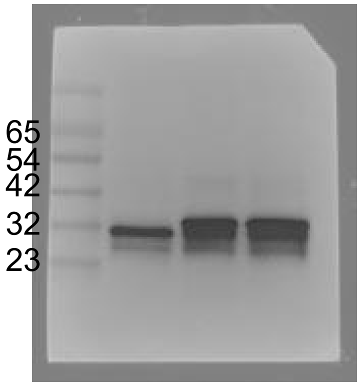


**β-ACTIN**

BCPAP

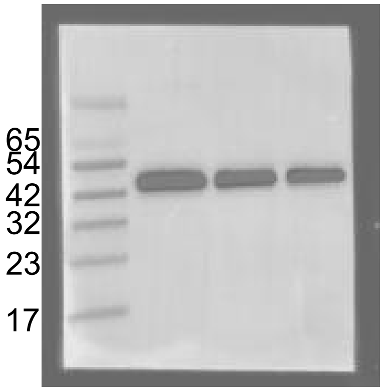


K1

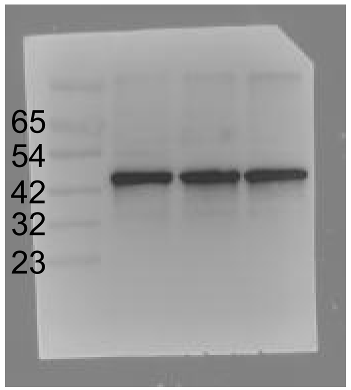

# Figure 4i

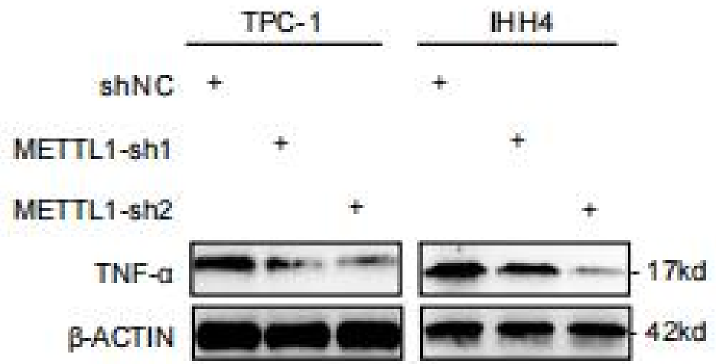


**TNF-α**

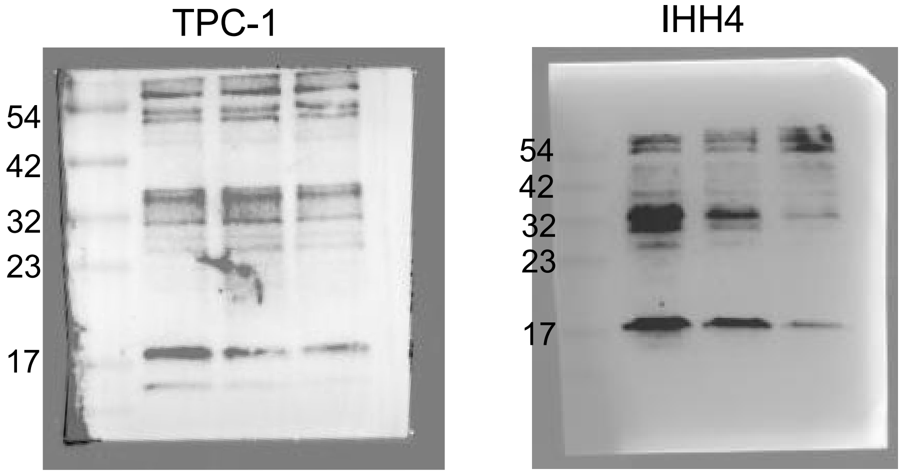


**β-ACTIN**

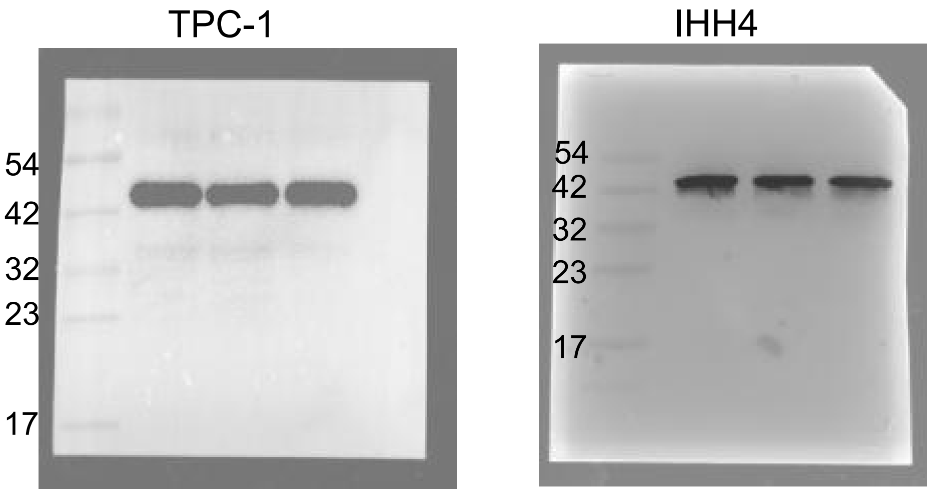

# Figure 5a

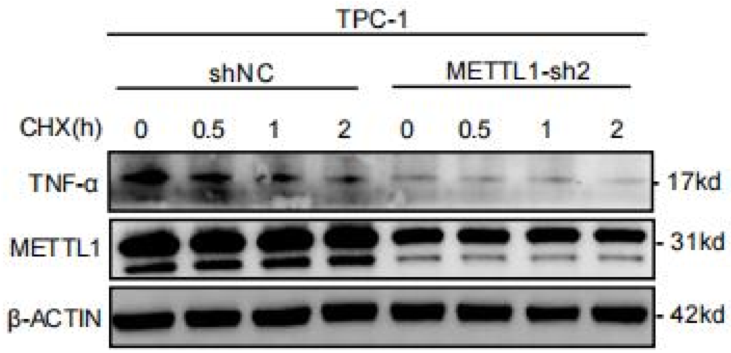


**TNF-α**

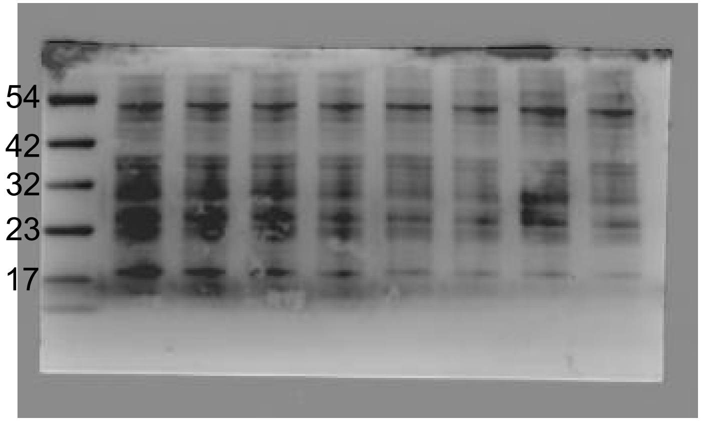


**METTL1**

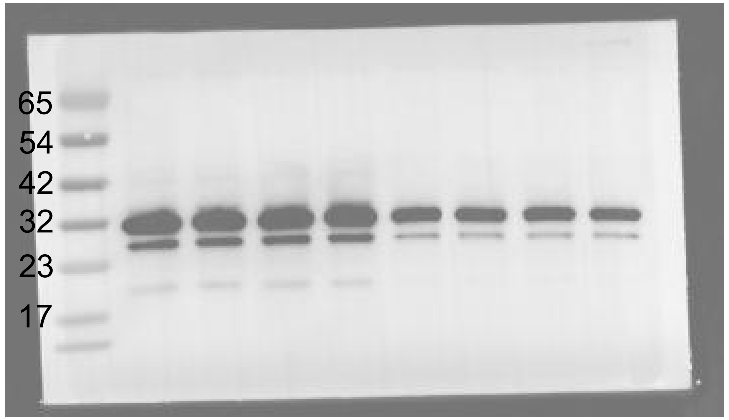


**β-ACTIN**

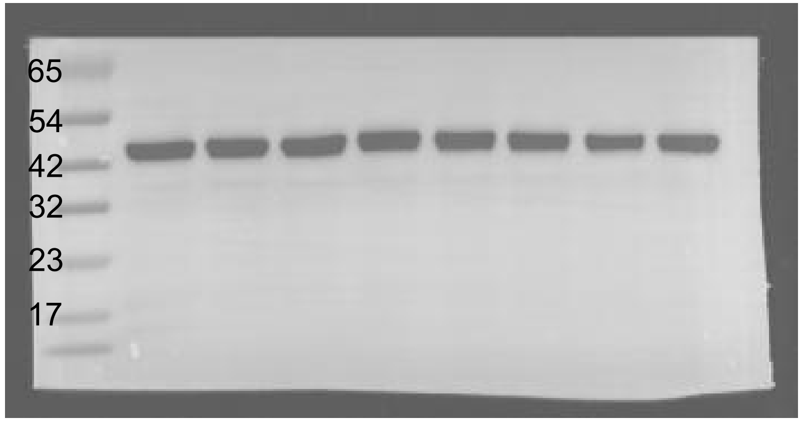

## Figure 5c

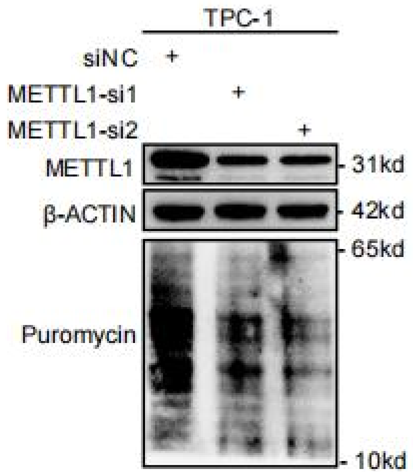


**METTL1**

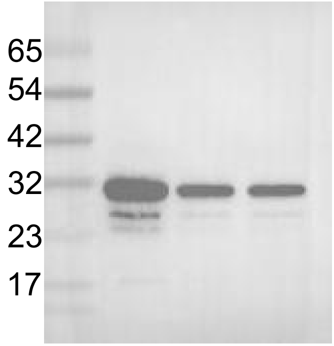


**β-ACTIN**

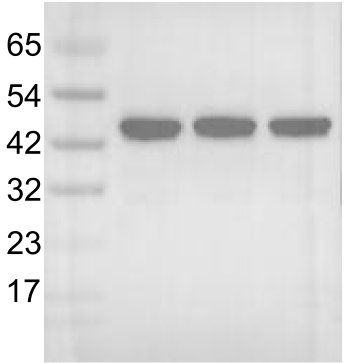


**Puromycin**

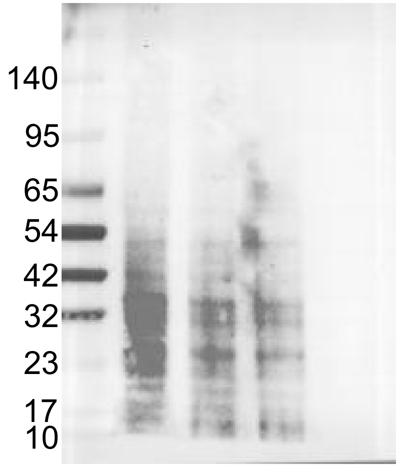

**Figure 5I**

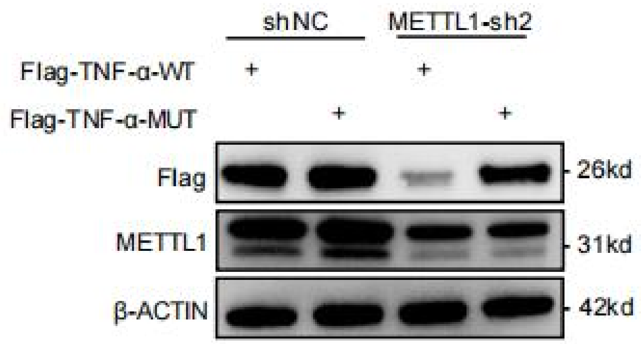
shNC
METTL1-sh2
Flag-TNF-α-WT
Flag-TNF-α-MUT
Flag
METTL1
β-ACTIN
26kd
31kd
42kd

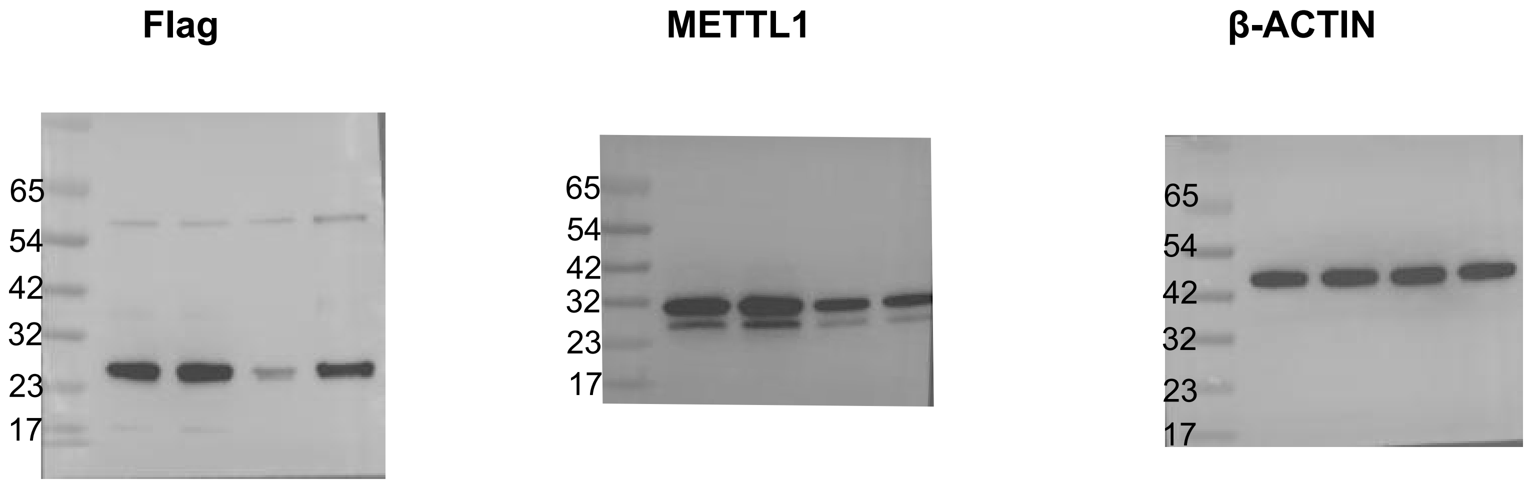
Flag
METTL1
β-ACTIN
65
54
42
32
23
17

## Figure 5n

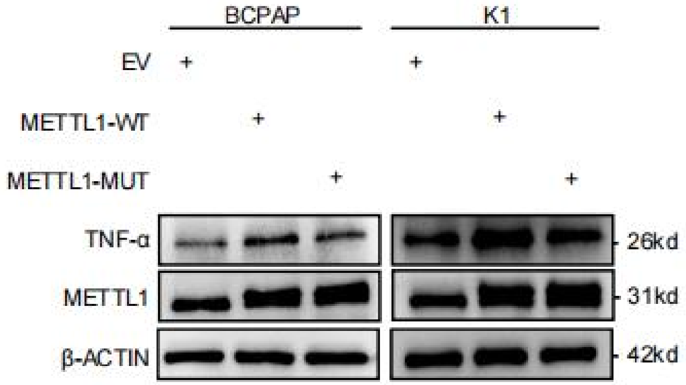


**TNF-α**

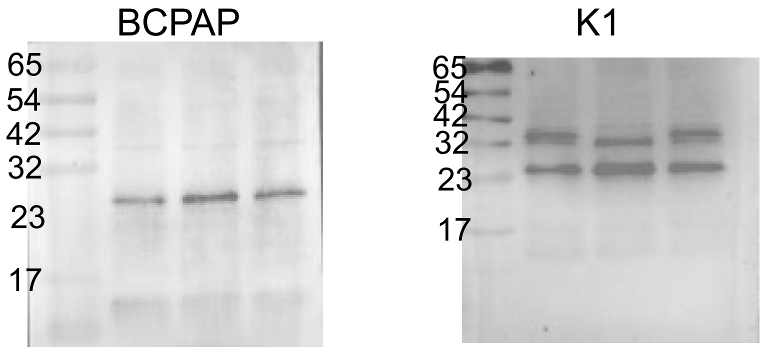


**METTL1**

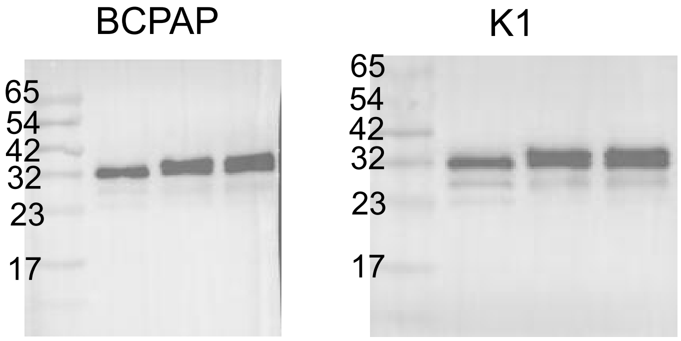


**β-ACTIN**

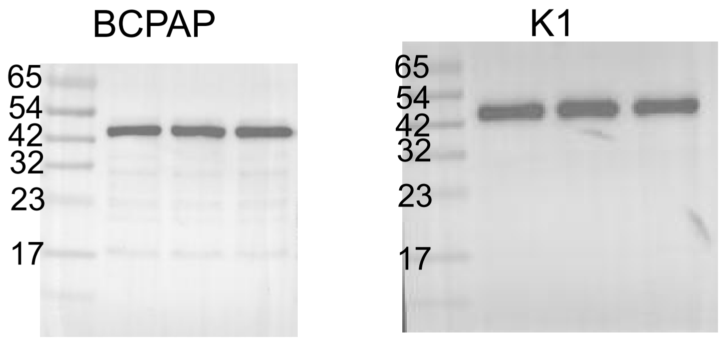

## Figure S2a

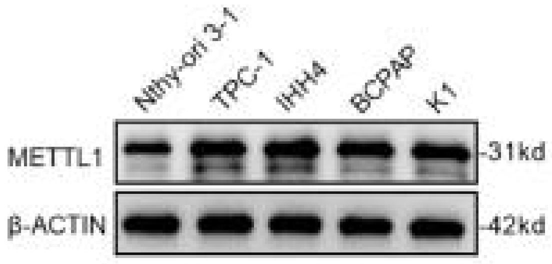


**METTL1**

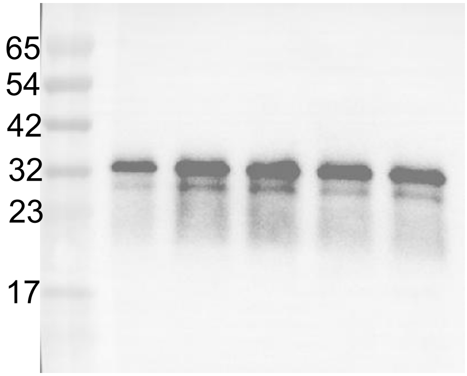


**β-ACTIN**

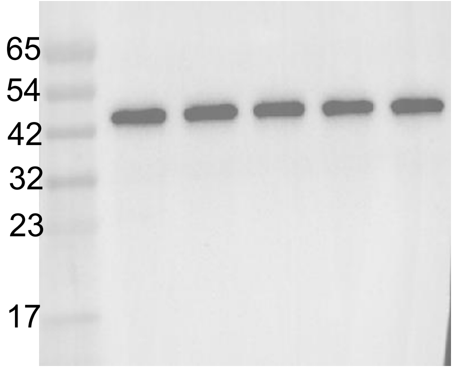

# Figure S4c

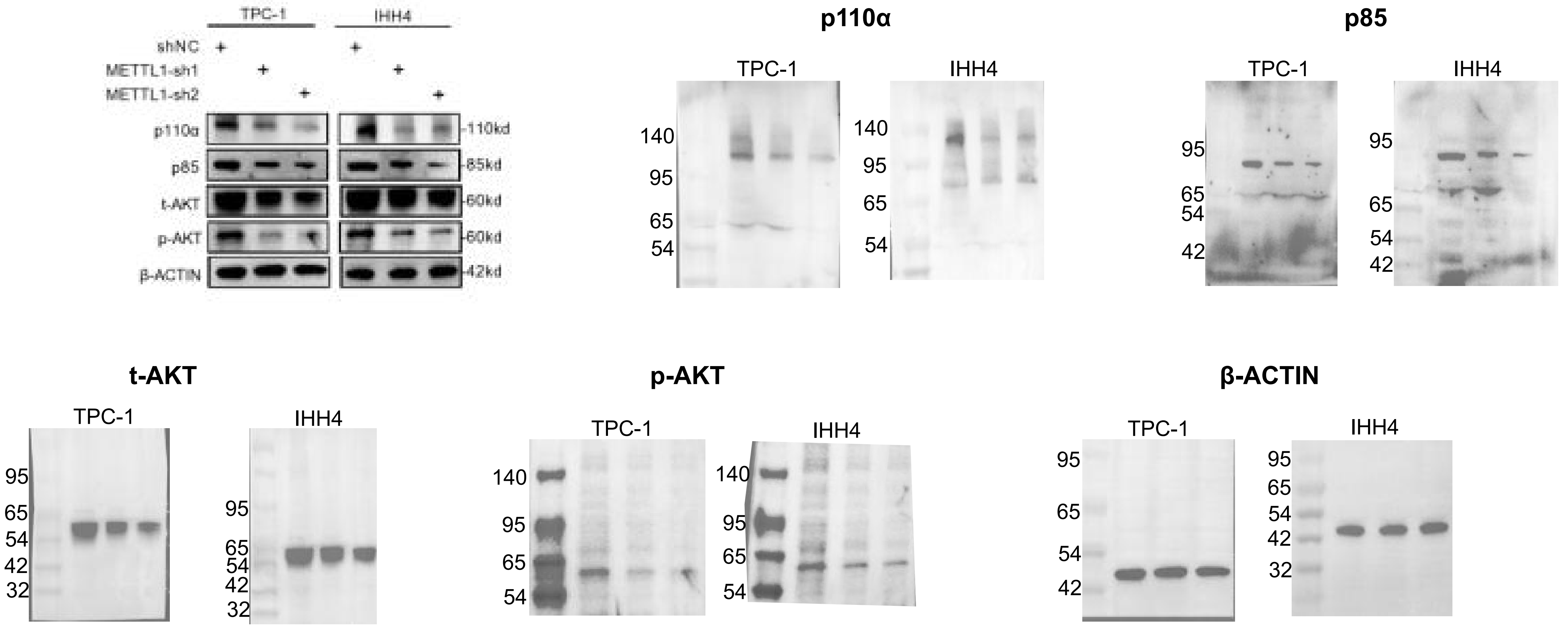

TPC-1
IHH4
shNC
METTL1-sh1
METTL1-sh2
p110α
p85
t-AKT
p-AKT
β-ACTIN
110kd
85kd
60kd
60kd
42kd
p110α
TPC-1
IHH4
140
95
65
54
p85
TPC-1
IHH4
95
65
54
42
t-AKT
TPC-1
IHH4
95
65
54
42
32
p-AKT
TPC-1
IHH4
140
95
65
54
β-ACTIN
TPC-1
IHH4
95
65
54
42
32
